# Towards outperforming conventional sensor arrays with fabricated individual photonic vapour sensors inspired by *Morpho* butterflies

**DOI:** 10.1038/ncomms8959

**Published:** 2015-09-01

**Authors:** Radislav A. Potyrailo, Ravi K. Bonam, John G. Hartley, Timothy A. Starkey, Peter Vukusic, Milana Vasudev, Timothy Bunning, Rajesh R. Naik, Zhexiong Tang, Manuel A. Palacios, Michael Larsen, Laurie A. Le Tarte, James C. Grande, Sheng Zhong, Tao Deng

**Affiliations:** 1 General Electric Global Research Center, Niskayuna, New York 12309, USA; 2 College of Nanoscale Science and Engineering, State University of New York, Albany, New York 12203, USA; 3 School of Physics, University of Exeter, Exeter, EX4 4QL, UK; 4 Materials and Manufacturing Directorate, Air Force Research Laboratory, Wright-Patterson AFB, Ohio 45433, USA; 5 Department of Bioengineering, University of Massachusetts Dartmouth, Dartmouth, MA 02747, USA; 6 State Key Laboratory of Metal Matrix Composites, School of Materials Science and Engineering, Shanghai Jiao Tong University, Shanghai 200240, People's Republic of China

## Abstract

Combining vapour sensors into arrays is an accepted compromise to mitigate poor selectivity of conventional sensors. Here we show individual nanofabricated sensors that not only selectively detect separate vapours in pristine conditions but also quantify these vapours in mixtures, and when blended with a variable moisture background. Our sensor design is inspired by the iridescent nanostructure and gradient surface chemistry of *Morpho* butterflies and involves physical and chemical design criteria. The physical design involves optical interference and diffraction on the fabricated periodic nanostructures and uses optical loss in the nanostructure to enhance the spectral diversity of reflectance. The chemical design uses spatially controlled nanostructure functionalization. Thus, while quantitation of analytes in the presence of variable backgrounds is challenging for most sensor arrays, we achieve this goal using individual multivariable sensors. These colorimetric sensors can be tuned for numerous vapour sensing scenarios in confined areas or as individual nodes for distributed monitoring.

## Abstract (web summary)

Individual vapour sensors often suffer from poor selectivity, which hinders their broad applicability. Here, Potyrailo *et al.* fabricate individual sensors inspired by the *Morpho* butterfly capable of selectively detecting vapours in mixtures and with a variable moisture background.

## Graphical abstract

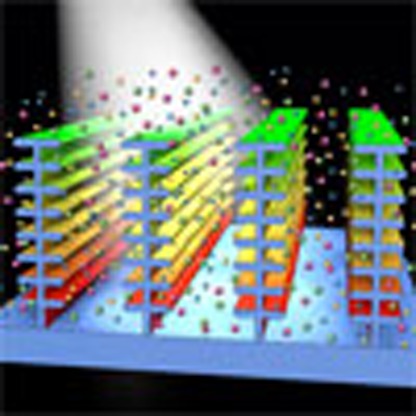

Selective detection of vapours in the presence of a complex background is needed in medical diagnostics, environmental surveillance, homeland protection and other applications. At present, ‘classic' analytical instruments based on gas chromatography, mass spectrometry and their hyphenated combinations^1^, are preferred, despite their relatively high power consumption, cost and size^2^,^3^. Vapour sensors could be a possible alternative to complex analytical instruments but existing sensors are applicable only when measurement conditions are simple and detection selectivity is not needed^4^. While sensitivity of sensors has been recently improved^5^,^6^, their selectivity remains the key challenge because available individual sensors cannot quantitate vapours in their mixtures and cannot operate in the presence of interferences^7^,^8^,^9^. Combining sensors into arrays^10^ has become a common compromise to mitigate poor selectivity of individual conventional sensors as shown in excellent studies with sensor arrays containing up to 65,536 elements^6^,^10^,^11^,^12^,^13^. However, the recognized limitation of arrays is the uncorrelated drift of each sensor^14^,^15^,^16^. Thus, stimulated by design principles found in nature that already have impacted many scientific fields^17^,^18^,^19^,^20^,^21^,^22^, we studied the iridescent wing scales of *Morpho* butterflies, found their unusually high-vapour response selectivity^23^ and discovered its origin^24^ with the goal of building individual vapour-selective sensors.

Here we developed design criteria for high-selectivity vapour sensing using *Morpho* scales as the bio-inspiration, fabricated such photonic sensors and compared their vapour-response selectivity with natural *Morpho* scales and several types of conventional sensor arrays. Our fabricated three-dimensional nanostructures not only selectively detect individual closely related vapours in pristine dry-gas conditions, similar to the natural *Morpho* scales and conventional sensor arrays, but also quantify these vapours even in mixtures in the presence of a variable moisture background. We have found that while natural *Morpho* scales and several types of conventional sensor arrays quantify individual closely related vapours well, their response degrade on attempts to quantify these vapours in mixtures and in the presence of water vapour interference. In contrast, we show that individual fabricated bio-inspired sensors outperform tested conventional sensor arrays in their vapour-response selectivity. The design principles for individual sensors introduced here complement existing sensing philosophy of sensor arrays by enhancing selectivity of individual sensors.

## Results

### Principle of vapour sensing with individual photonic sensors

**Figure 1:**
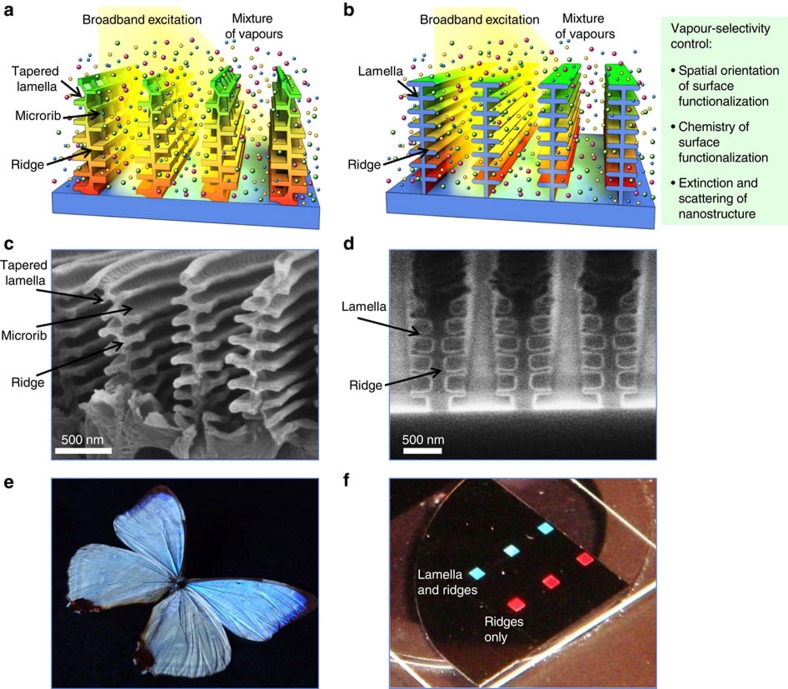
Design and fabrication of highly selective vapour sensors inspired by *Morpho* butterflies. (**a**) Schematic of the tree-like tapered structure of natural butterfly scales with its chemical gradient of surface polarity. (**b**) Schematic of the fabricated nanostructure and design criteria for vapour-selectivity control. (**c**,**d**) Scanning electron microscopy (SEM) images of *Morpho sulkowskyi* scales and fabricated six-lamellae nanostructures. (**e**,**f**) Iridescent colouration of *M. sulkowskyi* scales and fabricated nanostructures. Shown in (**f**) are six regions of nanostructures that were fabricated with and without lamella; each region was 2 × 2 mm. On illumination with a white light, three replicate regions of nanostructures with lamella reflected blue light while three other replicate regions of nanostructures without lamella (with only ridges) reflected red light.

The highly selective vapour detection using new individual sensors has become possible due to the careful sensor design that involved optical simulations, nanofabrication and extensive experiments (Fig. 1). The vapour-sensitivity of this sensor originates from two synergistic design aspects. First, the sensor is a resonant structure, designed to promote an enhanced vapour response compared with non-resonant sensors. It comprises a multilayer interferometric nanostructure with individual horizontal lamella supported through their middle by a vertical ridge. The periodic arrangement of these nanostructures also adds diffractive effects to the sensor response. Second, this resonator is designed to be an open-air structure to allow vapours to interact with all its regions. Depending on the material of the resonator, these interactions can lead to adsorption of vapours on the impermeable surfaces of the structure or to partitioning of vapours into a vapour-permeable material of the structure. Both types of interactions of vapours with the nanostructure lead to the controlled change of resonant conditions for the sensor.

The mechanism for the selective vapour response of these developed individual sensors involves spatially controlled interactions of different vapours with the fabricated nanostructures. These localized interactions with specific vapours are expressed in the corresponding regions of the reflectance spectra. This response to diverse vapours is described as multivariable sensing^24^,^25^, where an individual sensor has several partially or fully independent responses. The tree-like tapered structure of natural butterfly scales^17^,^26^ with its recently discovered^24^ chemical gradient of surface polarity (Fig. 1a) served as a guide to develop the design criteria for high-performance sensors (Fig. 1b).

The functional design of the sensor involves physical and chemical variables. The physical design uses not only optical interference and diffraction due to the fabricated periodic nanostructures, but also a small component of optical loss in the nanostructure. This loss gives rise to distinct signatures of reflectance spectra that are induced by the optical attenuation when light propagates between the top and bottom regions of the lamella stack. The chemical design uses spatially controlled nanostructure functionalization to promote distinct interactions of diverse vapours within the sensor.

We found that the tree-like tapered structure of natural butterfly scales (Fig. 1c) was not critical for vapour-response selectivity of nanofabricated sensors and their uniform length of the lamellae was adequate (Fig. 1d). Similar to blue iridescence of *Morpho* scales (Fig. 1e), we obtained strong structure-dependent iridescence from these fabricated nanostructures (Fig. 1f).

### Design of nanostructures for selective vapour sensing

**Figure 2:**
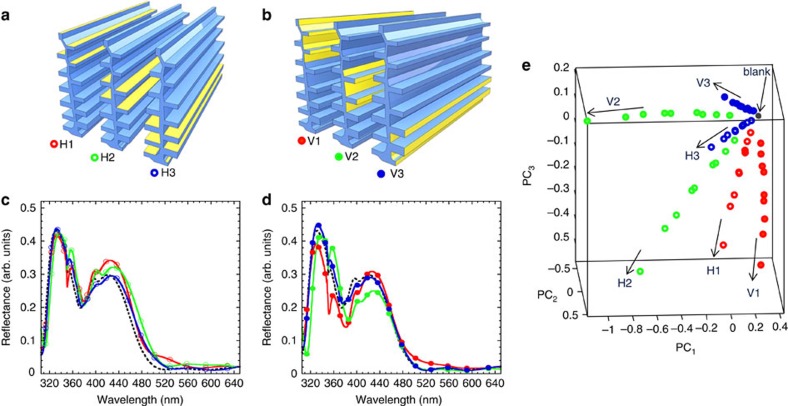
Results of modelling of vapour-response selectivity of the *Morpho*-like nanostructure. Employed in simulations were three vapours with *n*=1.3, 1.4 and 1.5 and their three vapour concentrations related to condensed liquid layers of thickness *d*=5, 10 and 15 nm. Adsorption of vapours on its (**a**) horizontal, H, and (**b**) vertical, V, regions of top, middle and bottom segments (sections 1–3, respectively) of the nanostructure. Reflectance spectra of adsorption of vapours on (**c**) horizontal and (**d**) vertical regions of the nanostructure. Shown are examples of adsorbed vapour with *n*=1.5 and *d*=15 nm. Dotted lines, spectra without adsorbed vapours (a blank with *n*=1, *d*=0 nm). (**e**) PCA scores plot of combined horizontal and vertical coverage of the nanostructure with three different vapours on top, middle and bottom segments. Contributions of PCs: 49.8%, 27.1% and 17.3%, for PC_1_–PC_3_, respectively, capturing 94.2% of the total variance.

**Figure 3:**
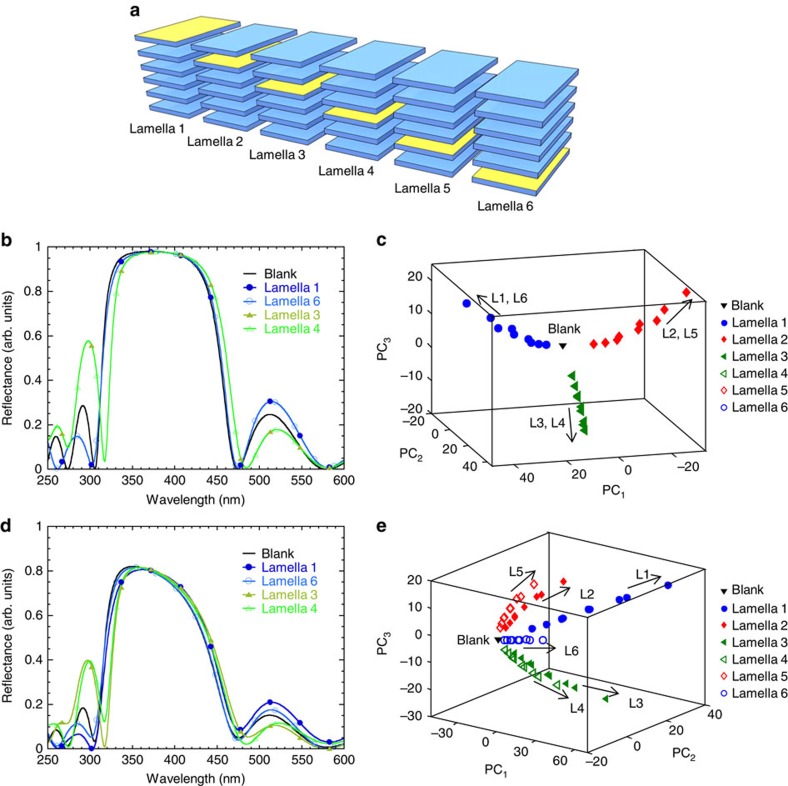
Results of modelling of vapour-response selectivity of a bio-inspired six-lamellae nanostructure with a variable extinction coefficient *k*. (**a**) Nanostructure design with a distribution of adsorbed vapours (*n*=1.3, 1.4 and 1.5; *d*=5, 10 and 15 nm) over individual lamella 1–6. (**b**,**d**) Examples of reflectance spectra with *k*=0 and 0.05, respectively. (**c**,**e**) PCA scores plots with *k*=0 and 0.05, respectively. Numbers of each arm in (**c**,**e**) correspond to the lamella (L) of the six-lamellae stack. Vapour discrimination in (**c**) was limited to confined effects of symmetrical lamella and resulted only in three resolved arms; contributions of PCs: 53.1%, 27.1% and 17.0%, for PC_1_–PC_3_, respectively, capturing 97.2% of the total variance. A complete vapour discrimination in (**e**) was due to resolved effects of non-zero lamella absorption, resulting in all six resolved arms; contributions of PCs: 47.4%, 22.0% and 13.5%, for PC_1_–PC_3_, respectively, capturing 82.9% of the total variance.

To develop design criteria for vapour-selective multivariable photonic sensors, we performed two sets of optical simulations. First, we investigated the ability to resolve optical responses from the horizontal and vertical regions of the nanostructures (Fig. 2). Second, we were interested to simplify the elaborate design of *Morpho* scales that includes tapered lamella profiles and spatially offset lamella^17^,^26^ to a more simple structure (Fig. 3) yet without the loss of the vapour-response selectivity observed with natural structures^23^.

Discrimination of vapours adsorbed on the horizontal and vertical regions on a *Morpho*-like nanostructure was explored by computing spectral responses of the structure with vapours adsorbed on its top, middle and bottom segments (Fig. 2a,b). Simulations over three sections were used as an approximation of a chemical gradient along the height of the structure that was originally discovered in natural *Morpho* scales^24^. The refractive index of the structure, *n*=1.56, and its extinction coefficient, *k*=0.06, were the same as in the natural *Morpho* architecture^26^. Adsorption of model vapours of solvents (with their *n*=1.3, 1.4 and 1.5) increased the effective refractive index of the sensor by replacing a fraction of air (*n*=1) by a fraction of a liquid analyte *(n*>1) with its gas-phase concentration-dependent thickness^27^. Thus, we simulated vapour concentrations as related to liquid layers of thickness *d*=5, 10 and 15 nm formed on the nanostructure (see Methods).

Reflectance spectra, simulated using finite element modelling (Fig. 2c,d and Supplementary Fig. 1a,c), illustrate two points of critical importance. First, the *Morpho*-like nanostructure had different spectra from the regions of the horizontal and vertical orientation, as desired for sensor selectivity. Second, the spectral differences from these regions had similar magnitudes, as desired for sensor sensitivity. The red-shift trends in the reflectance spectra on adsorption of vapours on the horizontal lamellar surfaces were expected based on classic multilayer interference effects, related to changes in the optical path *n* × *d* of the individual lamellar layers^28^. Interestingly, adsorption of vapours on vertically oriented regions also changed the respective optical paths of the nanostructure and the reflectance spectra.

The multi-wavelength nature of the reflectance spectra required the use of statistical tools to simplify spectral interpretation. To differentiate these spectra quantitatively, we used principal components analysis (PCA), a widely accepted technique for classification of multivariate data (see Methods for a detailed description). PCA results of the combined optical response of the *Morpho*-like nanostructure to vapour adsorption on horizontal and vertical regions illustrate data points grouped as six individual response directions or ‘arms' (Fig. 2e). These six response arms corresponded to vapours adsorbed on the top, middle and bottom segments of vertical and horizontal regions of the nanostructure. The nine data points in each response arm were related to nine reflectance spectra calculated for three vapours and their three adsorbed layer thicknesses related to their *n* × *d* product (optical path). All response arms started from a ‘blank', that is, a spectral data point produced from a spectrum of the nanostructure without adsorbed vapours (*n*=1, *d*=0 nm). All six modelled regions on the nanostructure were well resolved. For comparison, PCA was also performed for spectral responses from horizontal and vertical regions separately (Supplementary Fig. 1b,d).

These simulations provided strong support for the possibility of resolving vapours based on the spatially controlled interactions with horizontal and vertical regions of the nanostructure. Such interactions may be possible if diverse vapours are attracted to either horizontal or vertical regions of the nanostructure based on the differences in its chemical functionalization. Such ability should expand the range of vapours selectively detected by one sensor.

Next, we simplified the *Morpho-*like design from its elaborate tree-like nanostructure to a more simple and manufacturable nanostructure. We calculated reflectance spectra of a six-lamellae nanostructure on adsorption of the same model vapours (*n*=1.3, 1.4 and 1.5) and their vapour-concentration-dependent liquid layers (*d*=5, 10 and 15 nm) as employed with the *Morpho*-like nanostructure. Calculations of reflectance spectra of a six-lamellae nanostructure were performed using a Fresnel-based multilayer code (see Methods) on adsorption of vapours on each individual lamella (Fig. 3a). Unlike the use of only extinction coefficient *k*=0.06 of the lamellae given for the *Morpho* scales^26^, we were able to explore a range of extinction coefficients of the lamellae with *k*=0–0.2 (Supplementary Fig. 2).

We found that the selectivity of the sensor was strongly dependent on the extinction coefficient *k* of the lamellae. In the nanostructure with *k*=0, its reflectance spectra from different vapours and their vapour concentration-dependent liquid layers (*n* × *d*) adsorbed on lamella 1 were identical to those from vapours adsorbed on lamella 6 (Fig. 3b). Similarly, identical spectra were formed between lamella 2–5 and 3–4. Thus, vapour discrimination was limited to confined effects of symmetrical lamellae^29^ and resulted only in three resolved arms in PCA space (Fig. 3c). On the introduction of a small extinction in the lamellae material (*k*=0.05), unique reflectance spectra were associated with a specific lamella position of vapour absorption (Fig. 3d). A complete vapour discrimination was due to resolved effects of non-zero lamella absorption, resulting in forming of six distinct arms in PCA space (Fig. 3e).

Results of the variation of extinction coefficient *k* from 0 to 0.2 on the resulting sensor selectivity between each of the six lamellae (Supplementary Fig. 2) illustrate the key role of *k* in vapour-response selectivity. Identical reflectance spectra of the six-lamellae nanostructure having *k*=0 with vapours adsorbed on lamella 1 and 6, 2 and 5, 3 and 4 were due to the optical reciprocity effects^29^. However, on an increase of *k*≥0.05, the attenuation of light propagating in the six-lamellae nanostructure has provided different reflectance spectra for each lamella. Thus, we have found that the absence of the material extinction significantly reduced the vapour-selectivity effect, while the presence of even a small extinction provided such ability. This new insight into the critical role of the nanostructure extinction coefficient *k* on the selectivity of vapour response adds new diverse possibilities for the creation of individual sensors with tunable selectivity.

### Fabrication and surface modification of nanostructures

**Figure 4:**
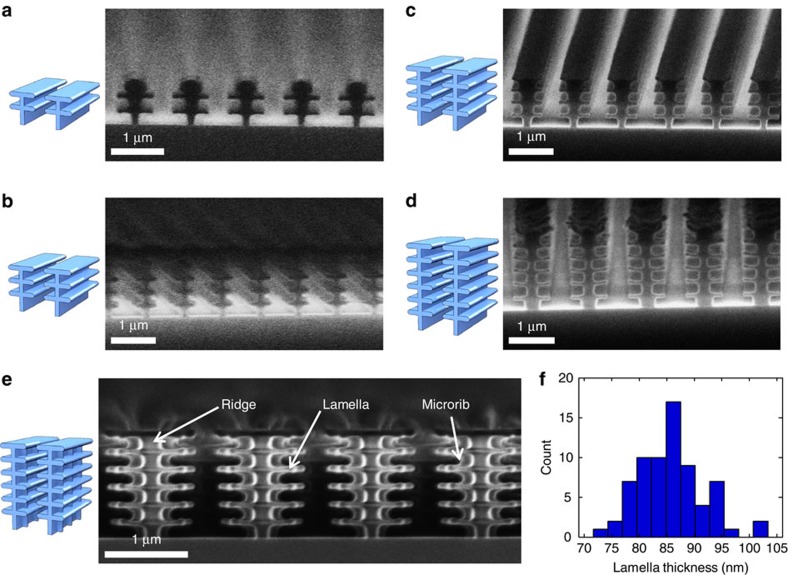
Fabricated photonic nanostructures with different number of lamellae for multivariable vapour sensing. (**a**–**d**) Schematics and SEMs of nanostructures with 2, 3, 4 and 6 lamellae, respectively. (**e**) Nanostructure with six lamellae and microribs. (**f**) Histogram of automated image analysis of lamellae thickness in six-lamellae nanostructures. Thickness measurements of lamella were taken 400 nm from the centre of the ridge. Resulting thickness of 86±6 nm (mean±1 σ, *n*=70) was a convolution of real thickness and image analysis uncertainty of±2 nm of edge determination.

Empowered by the results from performed optical simulations, we fabricated three-dimensional photonic nanostructures (Fig. 4). Fabrication of such nanostructures required control of the total height of the structure, number of lamellae and length of lamellae. We applied electron beam (e-beam) lithography for patterning of the alternating ∼100-nm thick layers of two types of positive tone photoresist materials poly(methyl methacrylate) (PMMA) and a copolymer of methyl methacrylate (MMA) and methacrylic acid (MAA). We further selectively removed the MMA/MAA copolymer using a binary solvent and formed intact PMMA lamellae (see Methods). Using this methodology, we fabricated nanostructures with different number of PMMA lamellae with its refractive index *n*=1.49 across the visible and with a negligible extinction coefficient (Fig. 4a-d). As an advanced demonstration, we fabricated structures with vertical microribs (Fig. 4e) similar to those observed in natural *Morpho* scales^23^. The careful control of fabrication conditions resulted in highly reproducible lamella thickness of 86±6 nm (Fig. 4f).

During our initial developments, we validated contributions of lamellae to reflectance spectra even for the minimal number of lamellae (Supplementary Fig. 3). Results of the optimization of e-beam dose for the fabrication of nanostructures with six lamellae are presented in Supplementary Fig. 4. By changing the spacing between the ridges of the nanostructures, we observed variable optical responses such as high-Q (quality factor) resonances (Supplementary Fig. 5). Large area nanostructures have been fabricated with a total area of >1 cm^2^ with high (>90%) reflectance and excellent spatial reflectance control (93.5±1.5% (mean±1 σ); RSD=1.6%) as shown in Supplementary Fig. 6.

Guided by the discovery of the chemical gradient in natural *Morpho* scales^24^, we further applied a localized chemical functionalization of the nanostructures. It was accomplished by the vapour-phase deposition of a silane followed by its exposure to an e-beam to form a chemical gradient on the surface of the fabricated nanostructures. Two types of silanes were explored, such as fluorine- and amine-terminated silanes, nonafluorohexyltrimethoxysilane (FS) and 3-aminopropyltrimethoxysilane, respectively (see Methods). A chemical gradient on the nanostructures was formed by their exposure to a 5 keV e-beam with a 100 μC cm^−2^ dose that was adequate to gradually remove the silane starting from the top of the structure (Supplementary Fig. 7). Amine- and fluorine-terminated silane coatings on the nanostructures are visualized in Supplementary Figs 8 and 9. Results of X-ray photoelectron spectroscopy analysis of these coatings before and after e-beam exposures are summarized in Supplementary Fig. 10 visualizing a controlled partial removal of coatings. Supplementary Note 1 includes a brief discussion on effects of fabrication tolerances and their relation to sensor calibration.

### Sensing performance of fabricated *Morpho*-inspired sensors

To evaluate response selectivity of fabricated nanostructured sensors, we designed vapour tests as four scenarios of increasing complexity, such as exposures to (1) individual vapours of diverse nature, (2) individual closely related vapours, (3) mixtures of closely related vapours and (4) mixtures of closely related vapours with a variable background.

For tests with individual diverse vapours, we selected benzene, methyl ethyl ketone, acetonitrile, methanol and water. Such exemplary vapours were selected to evaluate the ability of the developed individual sensors to discriminate between vapours of different nature. For tests with individual closely related vapours, we selected a challenging combination of vapours such as polar linear alcohols (model analytes) and water (interferent). Selection of these vapours allowed comparison of vapour responses of new fabricated multivariable sensors with responses of natural *Morpho* scales^23^,^24^ and different univariate resonant photonic sensors^28^,^30^,^31^,^32^,^33^. The addition of a more polar interferent (water vapour) to the mixture of alcohols makes quantitation of individual alcohols in mixtures even more challenging. However, such selection of the interferent has a significant practical impact because water vapour is the most abundant and high concentration interferent in ambient air^9^ and represents one of the biggest challenges for existing sensors^8^,^14^,^34^.

For all experiments, we used nanofabricated FS-functionalized sensors. In experiments with closely related vapours, for comparison we employed bare and functionalized natural *Morpho* scales, bare fabricated six-lamellae nanostructures and sensor arrays assembled using two types of conventional sensors. On the basis of our previous work with numerous types of sensor arrays^25^,^35^, we have selected quartz crystal microbalance (QCM) sensors and metal-oxide (MOX) sensors because of their diverse vapour-detection modalities that include both, vapour sorption and vapour-adsorption effects^4^,^8^. The QCM array was fabricated in house and had sensors coated separately with PMMA and FS films, the same materials as used for fabrication of photonic nanostructures. The MOX sensor array was a commercially available system with sensors broadly responsive to organic and inorganic gases (see Methods).

Fabricated sensors were initially tested for their vapour-response stability and reproducibility in reflectance *R* and differential reflectance *ΔR* modes. The *ΔR* spectra accentuated small spectral differences due to vapour response (see Supplementary Note 2 and Supplementary Fig. 11). Results of vapour exposures to 160 cycles are presented in Supplementary Fig. 12 illustrating sensor stability and measurement precision.

**Figure 5:**
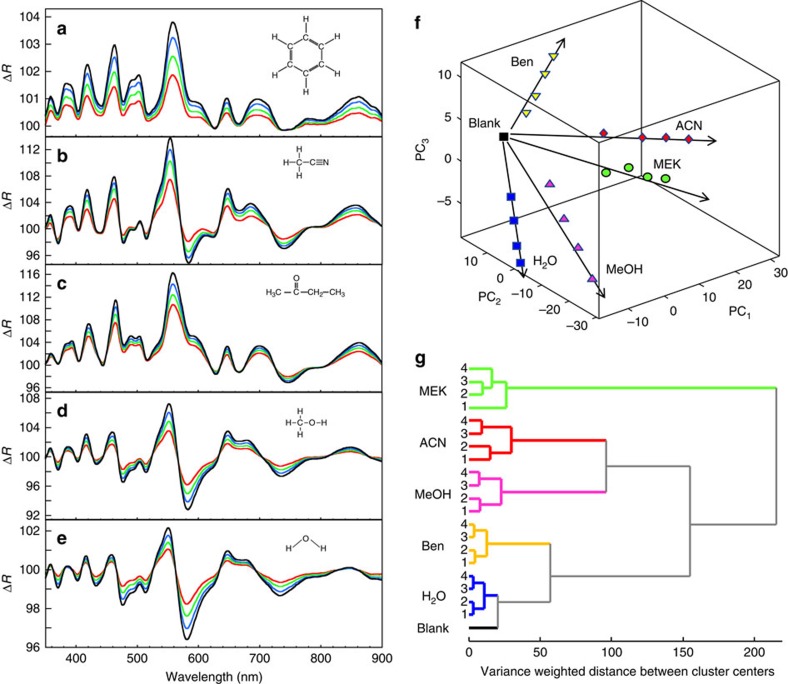
Diversity of optical interactions of a nanofabricated FS-functionalized multivariable sensor with vapours of different nature. (**a–e**) Δ*R* spectra on exposure to benzene (Ben), acetonitrile (ACN), methyl ethyl ketone (MEK), methanol (MeOH) and water (H2O) vapours. Concentrations of vapours: 0.05, 0.07, 0.09 and 0.11 *P*/*P*_0_ (labelled as red, green, blue and black lines, respectively), where *P* is vapour partial pressure and *P*_0_ is the saturated vapour pressure. (**f**) Discrimination of vapours using PCA. High contributions of the first three PCs (55.7%, 30.3% and 12.1%, for PC_1_–PC_3_, respectively, capturing 98.1% of the total variance) illustrate the high degree of data dimensionality even on exposure to only several vapours. (**g**) HCA dendrogram classification of Δ*R* spectra from five vapours at their four concentrations (1–4) using Ward minimum variance method.

Tests with five diverse vapours produced unique *ΔR* spectra (Fig. 5a-e) revealing diversity of optical interactions probed by the individual sensor. On performing PCA on these *ΔR* spectra, high contributions of the first PCs were achieved (55.7%, 30.3% and 12.1%, for PC_1_–PC_3_, respectively) illustrating the high data dimensionality from this sensor even on exposure to only several vapours (Fig. 5f and Supplementary Fig. 13). Data dimensionality should increase with the number of tested analytes in a well-designed sensor system^11^,^36^. We also performed a hierarchical cluster analysis (HCA, see Methods) because, in contrast to PCA, it classified the samples using raw Δ*R* spectra (Fig. 5g). Thus, both PCA and HCA independently showed clustering based on the nature of diverse vapours measured by the multivariable sensor.

Another approach to increase data dimensionality is to enhance the interaction strength between analytes and localized sensor regions by strongly ligating tested vapours at an expense of producing vapour dosimeters^37^. Chemical effects that are stronger than vapour sorption and adsorption will allow a wide range of chemical interactions and should facilitate an increased response dimensionality and further improve discrimination of vapours^38^.

**Figure 6:**
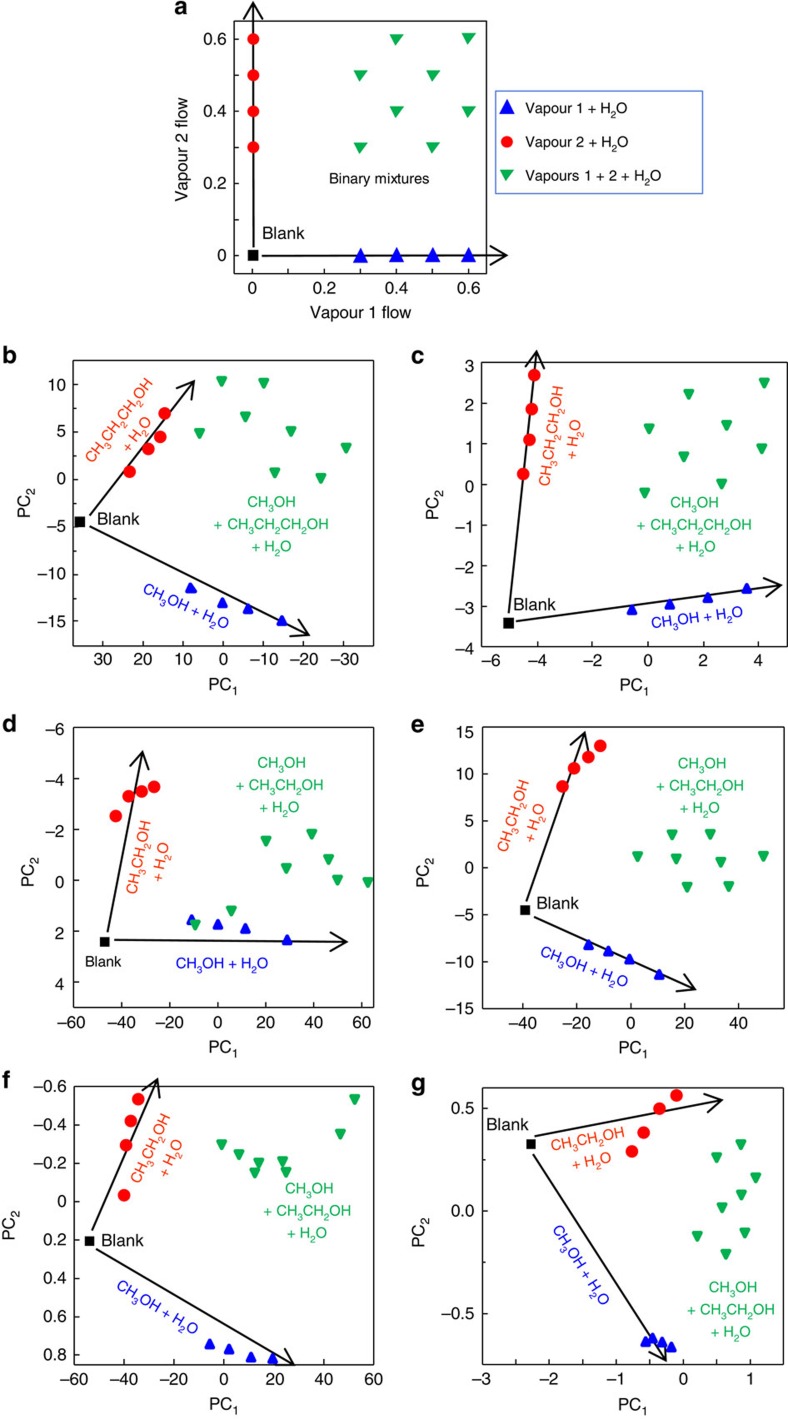
Ability of bio-inspired fabricated nanostructures, natural *Morpho* scales and QCM and MOX sensor arrays to quantify individual model vapours and their mixtures in the presence of water vapour background. (**a**) Designed map of concentrations of two individual model vapours and their binary mixtures mixed with water vapour for studies of responses of sensors, (**b**–**g**) PCA scores plots of responses of tested sensor systems to vapours and their mixtures. Sensors: (**b**) FS-functionalized natural *Morpho* scales and (**c**) FS-functionalized fabricated nanostructure—response to methanol and propanol in the presence of water. (**d**) bare and (**e**) FS-functionalized fabricated nanostructures—response to methanol and ethanol in the presence of water. (**f**) QCM and (**g**) MOX sensor arrays—response to methanol and ethanol in the presence of water. Concentrations of methanol, ethanol and propanol, 0.05, 0.07, 0.09 and 0.11 *P*/*P*_0_. Concentration of water vapour, 0.4 *P*/*P*_0_. Vapour labels: methanol (CH_3_OH), ethanol (CH_3_CH_2_OH), propanol (CH_3_CH_2_CH_2_OH) and water (H_2_O). Contributions of PCs of different sensors are summarized in Supplementary Table 2 and Supplementary Note 4.

Next, the fabricated sensing structures were exposed to closely related analyte vapours, followed by exposures to their binary mixtures and followed by adding a variable background of water vapour at 0.2 and 0.4 *P*/*P*_0_. Figure 6a depicts the layout of the experiments illustrating individual vapours at four concentrations and their additive combinations to form eight binary mixtures at different ratios. A time-dependent sequence of concentrations of two analyte vapours and an interferent water vapour is presented in Supplementary Fig. 14. The raw spectral and dynamic data, as well as quantitative correlation plots on exposure to individual vapours and their binary and ternary mixtures are presented in Supplementary Figs 15–20.

Initial vapour-response selectivity experiments with alcohols were performed with methanol and propanol because we achieved discrimination between these vapours in dry conditions using bare *Morpho* structures^24^. Using those results as a benchmark, we studied the discrimination of these vapours in the presence of humidity. Figure 6b,c compare responses of *Morpho* scales and fabricated nanostructures, both functionalized with FS silane. Although functionalized *Morpho* scales discriminated individual methanol and propanol vapours, their discrimination of individual vapours in mixtures was relatively poor. The experimental results (Fig. 6b) had relatively poor resemblance with the experimental layout (Fig. 6a) signifying that the contributions of individual vapours to the responses of *Morpho* scales to vapour mixtures were not purely additive. In contrast, the fabricated nanostructure had good discrimination between mixtures of vapours even in the presence of variable water vapour background. The PCA plot demonstrated an additive response of vapours in their mixtures even at the highest tested humidity (Fig. 6c).

Supplementary Fig. 16 demonstrates the details of the ability of the fabricated sensor to quantify two individual model vapours and their binary and ternary mixtures when blended with water vapour at different levels. Observation of sensor response at different wavelengths provided an important insight into the multivariable nature of the sensor. At certain wavelengths (for example, 559 nm), the sensor had a minimal response to water, whereas at other wavelengths (for example, 504, 585, 723 nm), the response to water was strong and additive with responses to analytes. Also, at certain wavelengths (for example, 559, 723 nm), the sensor responded to two analytes in the same direction, whereas at other wavelengths (for example, 504, 585 nm), responses to two analytes were in the opposite directions. Further, dynamic responses on propanol and methanol exposures had distinct recovery signatures at different wavelengths—for example, fast and slow recovery at 559 and 723 nm, respectively, and a non-monotonic recovery from propanol/methanol mixtures at 504 nm. This diversity of dynamic signatures illustrated several interaction mechanisms of vapours with different regions of the functionalized nanostructure.

These diverse and additive responses allowed a multi-wavelength regression such as partial least squares (PLS, see Methods) to be applied to the Δ*R* spectra to independently quantify methanol and propanol in their binary mixtures, and in ternary mixtures with water vapour at 0.2 and 0.4 *P*/*P*_0_ (see Supplementary Fig. 17).

Further, the sensors were tested with the challenging combination of alcohols, that is, methanol and ethanol, which are the first and second linear alcohols in their homologous series. These studies were also carried out with individual vapours, their binary mixtures, and ternary mixtures when blended with variable water vapour background. Tests were performed using bare and FS-functionalized fabricated nanostructures. *Morpho* scales were excluded because of their relatively poor response to methanol/propanol mixtures. Comparison of responses of the bare (Fig. 6d) and the FS-functionalized (Fig. 6e) nanostructures revealed good discrimination of methanol and ethanol vapours by the FS-functionalized nanostructure even when vapours were mixed with water vapour. Supplementary Figs 18–20 illustrate the ability of the FS-functionalized nanostructure to quantify methanol and ethanol at a variable water vapour background. Supplementary Table 1 and Supplementary Note 3 provide additional details on the sensor ability for quantitation of individual vapours in mixtures.

Finally, a comparison of responses of the FS-functionalized nanostructure with QCM and MOX sensor arrays to methanol and ethanol vapours, their binary mixtures and ternary mixtures with water vapour was performed. The developed multivariable sensor (Fig. 6e) resolved well individual vapours, binary and ternary mixtures and demonstrated a better response linearity versus both sensor arrays (Fig. 6f,g). Performance of each sensor system was further analysed based on the contributions of individual PCs of the PCA models (see Supplementary Table 2 and Supplementary Note 4). Although the MOX sensor array had the response dimensionality larger than that of the FS-functionalized nanofabricated structure, the latter had better response linearity.

## Discussion

We view this study as the introduction of new design and fabrication principles of individual multivariable sensors toward outperforming selectivity of conventional sensor arrays and approaching selectivity of ‘classic' and micro-fabricated gas chromatography and mass spectrometry analytical instruments. These instruments are often inconvenient, even with the reduced carrier gas or vacuum demands^39^, but are an unavoidable alternative when existing sensors cannot meet the monitoring requirements because of their poor selectivity. However, there are numerous application scenarios when high-selectivity advantages of even micro-fabricated gas chromatography and mass spectrometry instruments would be cancelled by specific application requirements. Examples of such applications include chemical surveillance in public places using unobtrusive self-contained sensor nodes of wireless sensor networks, home health care, workplace monitoring using wearable sensors, monitoring in subsea or down-hole oil and gas production, monitoring in harsh environments and others^40^,^41^,^42^.

Our design criteria for selective vapour sensing using individual photonic nanostructures (Fig. 1b) involve equally important physical and chemical control. Physical control can be achieved by the nanostructure geometry and physical mechanisms of light loss in the nanostructure. Geometry of the nanostructure involves interferometric lamella and their supporting ridges resulting in a highly ordered hierarchical photonic design. Vertical ridges serve as spacers to provide a high refractive index contrast (air gap) between lamella and to induce the ability for vapours to interact with the vertical regions of the nanostructure for vapour-selectivity enhancement. The tapered tree-like design of natural *Morpho* nanostructures with their spatially offset lamella^17^,^26^ was found to be not important for the selective vapour response, thus simplifying sensor design. We have found that even a slight controlled loss of light in the nanostructure (by material extinction and/or scattering) resulted in a desired enhancement of diversity of reflectance spectra on localized vapour adsorption (see Supplementary Note 5 for more details).

Chemical control can be achieved by gradient functionalization of the whole photonic nanostructure or individual sensing lamella. Diverse functionality can be also introduced into the horizontal and vertical regions of the nanostructures through the multi-layer e-beam approach reported in this work with further modified chemical composition of individual layers^43^. Multilayer nanotransfer printing can also be used to produce nanostructures with multiple diverse materials^44^ to complement the range of materials available from e-beam fabrication. This broad materials selection should allow tuning the response selectivity of sensors across the wide range of gaseous species. It should be also possible to achieve spectral discrimination from not only horizontal and vertical regions but also regions fabricated at other angles. Such ability for the superior selectivity could be achieved using different gradient functionalization techniques^45^.

Although this study was focused on the selectivity enhancement, if needed, a significant improvement in sensitivity of vapour-response of our multivariable sensors is also possible from our current part-per-million limits of detection (see Supplementary Table 3 and Supplementary Note 6). Sensor sensitivity was not the focus of this study because design criteria for sensitivity enhancement are well established. For example, physical means to achieve sensitivity improvement can include increasing the number of fabricated lamella, inducing the high-Q resonances in reflectance spectra by the careful ridge-to-ridge spacing control (see Supplementary Fig. 5), and operating the sensor in a stimulated emission regime^46^. Chemical means to achieve sensitivity improvement can include forming hierarchical substructures with higher surface area using different porogens to create mesoporous voids in the nanostructure and selection of lamella material with a high partition coefficient to vapours of interest^9^,^47^,^48^.

The knowledge of the design principles of these photonic multivariable sensors brings a fresh perspective and opportunities to build individual highly selective sensors. In such sensors, their spectral responses can be locally modulated by the refractive index and extinction coefficient of the structure^26^ and could benefit from an enhanced extinction in the violet spectral range^49^. Besides extinction, scattering could also contribute to increased diversity of reflectance in new designs. These sensing responses may become possible by the careful selection of *n*, *k* and scattering of the nanostructured lamellae. Because these sensors detect local changes in the optical path *n* × *d*, they can be used not only for gas-phase but also for liquid-phase measurements, for example for label-free biological sensing.

The photonic nanostructure design concept introduced here on a planar wafer can be further implemented in different configurations (for example, chips^50^ or waveguides^51^). Besides the approach described here to fabricate nanostructured multivariable sensors, other approaches are possible based on variations of focused ion-beam^52^ and e-beam^53^ lithography, direct etching^54^, etching/photolithography^55^, and other known methods. Important aspects for acceptance of these individual sensors will be their manufacturability^56^ and stability^15^. The detailed tuning of selectivity of our developed multivariable sensors to different classes of vapours, evaluation of the limitations of these new sensors in quantitative measurements of an increasing number (for example, 10–100) of analytes, manufacturing cost, stability of individual sensors versus sensor arrays, and effects of tolerances and variations in geometry of fabricated nanostructures on selectivity is the focus of our ongoing studies.

Results of this study should also inspire new fundamental research and technological innovations in the areas of photonics, materials science, chemistry and nanomanufacturing. From the fundamental science perspective, tuning of the hierarchical geometry and chemical functionality of the fabricated resonant nanostructures and the optical constants of the nanostructures' materials can be used to refine their optical resonance strength, spectral purity of reflectance and polarization properties. Such approach opens new insight in design and engineering of functional materials with hierarchical structures. Technologically innovative applications can include nanostructured reflectors with and without chemical modifications and can range from photonic security tags to encrypted communication devices, high-performance gas separators and many others.

## Methods

### Design and fabrication of bio-inspired nanostructures

Layouts of nanostructures were designed using a Layout editor (Juspertor UG, Germany). Nanostructures were fabricated by e-beam lithography using two positive tone e-beam photoresist materials^53^,^57^ PMMA and a copolymer of MMA and MAA obtained from Microchem (Newton, MA, USA). Photoresists were spin coated on silicon wafers using a spin coater (Model CEE 200, Brewer Science, Rolla, MO, USA) to form ∼100-nm thick layers. Wafers were soft baked at 150 °C for ∼1 min. The steps of spin coating and baking were repeated until desired number of layers was produced. Exposures were performed using an e-beam lithography system (Model VB300, Vistec Lithography B.V., the Netherlands) operating at 100 keV. Patterning was performed using a spot size of 20 nm with a beam step size of 20 nm. An e-beam dose of 800–1000 μC cm^−2^ was selected to pattern up to six bi-layers of PMMA/MMA–MAA which formed six lamellae along the penetration depth of the e-beam. A full dose was used to clear both PMMA and MMA–MAA copolymer layers and to form spaces between the structures. To form lamella by clearing only the copolymer layer, ∼15% of the full dose was needed. Pattern write time at these conditions was 3–4 min mm^−2^. Exposed wafers were developed for ∼1 min in a developer solution (methyl isobutyl ketone and isopropanol mixture at a 1:3 ratio). The developer was carefully blown using a dry nitrogen gun with a minimal shear from developer that otherwise was deforming the structures.

### Functionalization of bio-inspired nanostructures

Fabricated nanostructures were coated with monolayers of a fluorine-terminated silane (FS) or an amine-terminated silane (3-aminopropyltrimethoxysilane) using a vapour deposition process. Silanes were purchased as liquids from Sigma-Aldrich (St Louis, MO, USA). Several microliters were dispensed on a glass slide and put in an enclosed chamber with fabricated nanostructures and incubated in an oven at 65 °C for 1 h. A chemical gradient on the nanostructures was formed by their exposure to a 5 keV e-beam with a ∼100 μC cm^−2^ dose that was found to gradually remove the silane starting from the top of the structure.

### *Morpho* butterfly samples

Dried *Morpho sulkowskyi* butterflies were obtained from Butterfly Utopia (Brooklyn, NY, USA). Samples were used as received or were functionalized with FS or 3-aminopropyltrimethoxysilane silanes using the same method as for functionalization of nanostructures.

### Scanning electron microscopy characterization

A field emission scanning electron microscope (SEM; Model S4800, Hitachi, Tokyo, Japan) was used for SEM imaging of the patterned samples. Minimal beam voltage (1 keV) and current (<1 nA) were used for high-resolution imaging. At these conditions, SEM imaging of fabricated nanostructures was performed without deposition of any thin layers of a conducting film.

### Transmission electron microscopy characterization

The cross-sections of fabricated nanostructures were prepared for transmission electron microscopy (TEM) characterization by focused ion beam (FIB) milling using a SEM/FIB system (Model Nova 200 Nanolab, FEI Company, Hillsboro, OR, USA) with an *in-situ* nanomanipulator (Model Omniprobe, Oxford Instruments, Abingdon, UK). Unnecessary exposure of the fabricated structure to the Ga ions during the FIB process due to instability of PMMA was minimized. X-ray maps were produced using a Tecnai F20 TEM/STEM (FEI Company) microscope operated at 200 kV with an X-ray spectral microanalysis system (Model NORAN System 7, Thermo Electron Scientific Instruments, Madison, WI, USA) with a 400 mm^2^ Si(Li) crystal, 138 eV energy resolution and a 0.18 steradian sold angle.

### Auger electron spectroscopy characterization

The Auger electron spectroscopy (AES) data was collected using a scanning Auger nanoprobe (Model PHI 700Xi, Physical Electronics, Chanhassen, MN, USA) using a 20 kV, 3 nA incident e-beam. AES maps were obtained by stepping the e-beam over a 256 × 256 array within the field of view. The beam was held for 5 ms at each step and the cycle was repeated 20 times. Samples were generated using a focused ion beam lift out using a method similar to the method used for TEM. The sample thickness was ∼1 μm instead of the 0.1 μm typically required for TEM. Before AES, the surface was sputtered with a 2 kV, 1 nA argon ion beam to reduce surface contamination.

### Vapour exposures

Different concentrations of vapours were produced using a built-in-house computer-controlled vapour generation system (total gas flow of 450 mL min^−1^). Vapours were delivered to nanofabricated structures or to butterfly wings by a 3-mm diam. tubing at ∼45° from the normal to the sample. For vapour exposures of nanofabricated structures, they had a 2 × 2 mm^2^ area and were oriented to achieve the maximum intensity of the reflected peak. For vapour exposures of butterfly wings, flat regions on the wings were secured with a metal washer (∼5-mm inner diam.). The vapour flow was arranged parallel to the ridges on the scale and to deliver the flow in the direction from the scale socket to its distal edge. A hydration step between switching to different vapours was used to speed up the recovery of *Morpho* wing samples to its baseline and was carried out by exposing the wing sample to humid carrier gas (0.35 *P*/*P*_o_). The hydration step was not needed during testing of nanofabricated structures.

### Reflectance measurements

A halogen light source (Model HL-2000-FHSA Mikropack, Ocean Optics, Dunedin, FL, USA) and a 16-bit spectrograph (Model QE65000, Ocean Optics) were used for reflectance measurements. The spectrograph was controlled by a written-in-house LabVIEW program (National Instruments, Austin, TX). The six-around-one fibre-optic reflectance probe was positioned at ∼30° from the normal to the sample surface. The size of the illuminated area was ∼2-mm diam. The distance between the sample and the probe was ∼2 mm. For enhanced reproducibility of measurements, the initial maximum reflectance in the scope mode and under a flow of a blank gas was ∼50,000 counts. In vapour-exposure experiments, differential reflectance spectra Δ*R* were measured (as % of reflectance change as compared with the blank carrier gas, see Supplementary Note 2). During sensor testing, the tip of the fibre-optic probe was also exposed to vapours. However, adsorption of vapours on the fibre alone produced no detectable effects as tested by illuminating a Si wafer, collecting reflected light and exposing both the wafer and the fibre tip to different vapours. The fibre-optic probe alone did not contribute to reflectivity spectra for two reasons: (1) unlike nanostructured sensors, it did not contain open-air resonant features that enhance vapour-adsorption effects; (2) adsorbed vapours did not generate an interference pattern in the visible spectral range where the measurements were performed.

### Optical modelling

Finite element modelling was used to calculate the total light reflectance from *Morpho-*like photonic structures. A unit cell containing a geometric representation of the butterfly's nanostructure was created from a TEM image. Reflectance from an infinite array of unit cells, with 550-nm ridge spacing, was then simulated for nanostructures without and with horizontal or vertical vapour overlayer conditions. Simulation results were obtained for light at normal incidence to the nanostructure, with the electric-field polarized parallel to the structure's ridge. To ensure the accuracy of finite element modelling calculations, the mesh parameters were optimized to obtain a converged solution for the rendered *Morpho* geometries (with and without localized model vapour adsorption). Reflectance of multi-lamella stacks with varying extinction coefficient *k* was calculated using a written in-house recursive Fresnel-based multilayer code. Calculations were carried out in MATLAB (The MathWorks, Inc., Natick, MA, USA). A six-lamellae ‘cuticle—air' stack of alternating refractive index layers had 55-nm thick lamella (*n*=1.56) and 100-nm thick air gaps (*n*=1). Values of condensed liquid layers (*d*=5, 10 and 15 nm) were selected based on the achievable spectral resolution of simulations and literature-reported thickness of condensed liquid layers on porous films^58^,^59^. In all simulations, the refractive index of the structure was not affected by adsorption of vapours.

### Conventional sensor arrays

A QCM sensor array was built using two 10-MHz quartz transducers (International Crystal Manufacturing, Oklahoma City, OK, USA). Individual QCMs were coated with PMMA and FS films. The response of the QCM array was measured using a network analyzer (model E5062A, Agilent Technologies, Inc. Santa Clara, CA) using a wireless proximity readout^60^. A sensor system containing an array of MOX sensors incorporated into a low-dead volume gas flow cell was obtained from Airsense, Germany. Four MOX sensors were selected for their response to polar organic compounds, nonpolar organic compounds and aromatic compounds^61^.

### Analysis of spectral and sensor array data

Analysis was carried out using KaleidaGraph (Synergy Software, Reading, PA, USA) and PLS_Toolbox Software (Eigenvector Research, Inc., Wenatchee, WA, USA) operated with MATLAB (The Mathworks Inc., Natick, MA, USA). Multivariate data processing (PCA, PLS and HCA) was carried out in MATLAB.

### Principal components analysis

PCA is a robust unsupervised pattern recognition tool for classification of multivariate data^62^. PCA reduces a multidimensional data set for its easier interpretation by calculating orthogonal principal components that are oriented in the direction of the maximum variance within the data set. The first principal component contains the highest degree of variance, and other PCs follow in the order of decreasing variance. Thus, the PCA concentrates the most significant characteristics (variance) of the data into a lower dimensional space. The distribution of data points in the PCA plot allows the visualization of relations between the original reflectance spectra after their mean-centering or auto-scaling. The origin of PCA plots (for example, the (0,0) point in each of the PCA plots) is the mean center of a particular data set and is not expected to coincide with a data point from a blank.

### Hierarchical cluster analysis

This technique classifies samples using complete Δ*R* spectra. The HCA dendrogram was obtained using Ward's method that shows the Euclidean distance between the trials^11^,^36^. The Ward's method is a minimum variance method, which takes into consideration the minimum amount of variance between the samples and analytes to define a cluster.

### Partial least squares

To quantify concentrations of individual vapours in mixtures, we applied multivariate regression modelling using PLS with second-order polynomial terms^62^. The PLS determines correlations between the independent variables (wavelengths) and the sensor response by finding the direction in the multidimensional space of the sensor response that explains the maximum variance for the independent variables. The key outputs of the developed multivariate models were the root mean square error of calibration and the root mean square error of cross-validation.

## Additional information

**How to cite this article:** Potyrailo, R. A. *et al.* Towards outperforming conventional sensor arrays with fabricated individual photonic vapour sensors inspired by *Morpho* butterflies. *Nat. Commun.* 6:7959 doi: 10.1038/ncomms8959 (2015).

## Supplementary Material

Supplementary Information Supplementary Figures 1-20, Supplementary Tables 1-3, Supplementary Notes 1-6 and Supplementary References.
