# Supplementary material for: Towards outperforming conventional sensor arrays with fabricated individual photonic vapour sensors inspired by Morpho butterflies
Source: Nat Commun. 2015 Sep 1;6:7959. doi: 10.1038/ncomms8959 (PMC4569698; doi:10.1038/ncomms8959)
Supplement: Supplementary Information — Supplementary Figures 1-20, Supplementary Tables 1-3, Supplementary Notes 1-6 and Supplementary References. [file ncomms8959-s1.pdf]

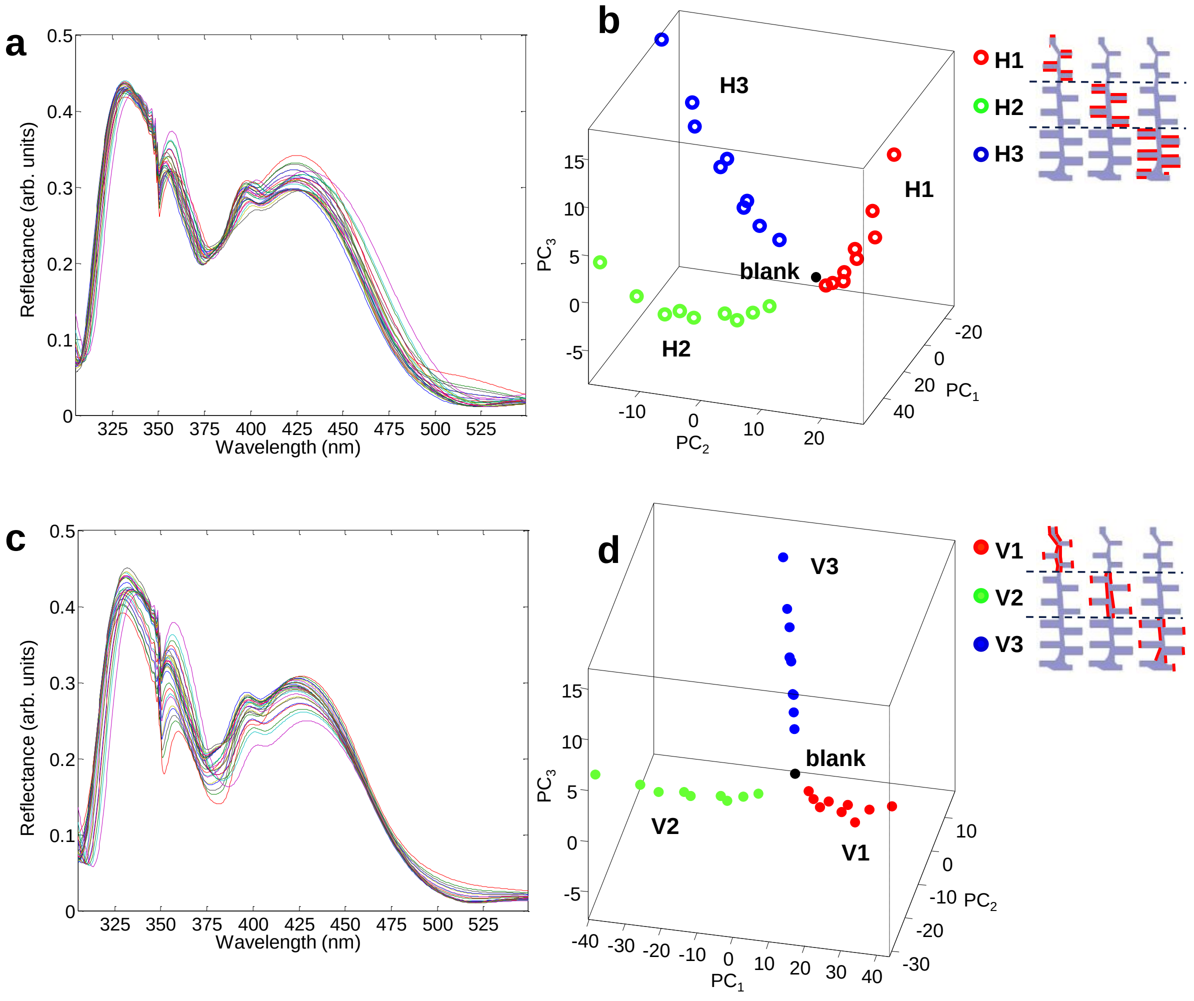

Supplementary Figure 1. Details of simulation results of vapour-response selectivity of the *Morpho*-inspired nanostructure based on spatially-controlled adsorption of vapours onto its horizontal (H) and vertical (V) regions of top, middle, and bottom segments of the nanostructure (segments 1 – 3, respectively). (a) 28 reflectance spectra and **(b)** PCA scores plot of vapour adsorption onto horizontal regions of the top, middle, and bottom segments of the nanostructure. Contributions of PCs: 56.8, 29.0, and 8.9%, for PC_1 – PC_3, respectively. **(c)** 28 reflectance spectra and **(d)** PCA scores plot of vapour adsorption onto vertical regions of the top, middle, and bottom segments of the nanostructure. Contributions of PCs: 63.5, 29.1, and 5.6%, for PC_1 – PC_3, respectively. Each data point in **(b, d)** is a PCA-processed spectrum related to the changes in $n \times d$. Employed in simulations are three vapours with n = 1.3, 1.4, and 1.5 and their three vapour concentrations related to condensed liquid layers of thickness d = 5, 10, and 15 nm. Blanks in (**b, d**) are spectra without adsorbed vapours (n = 1, d = 0 nm).

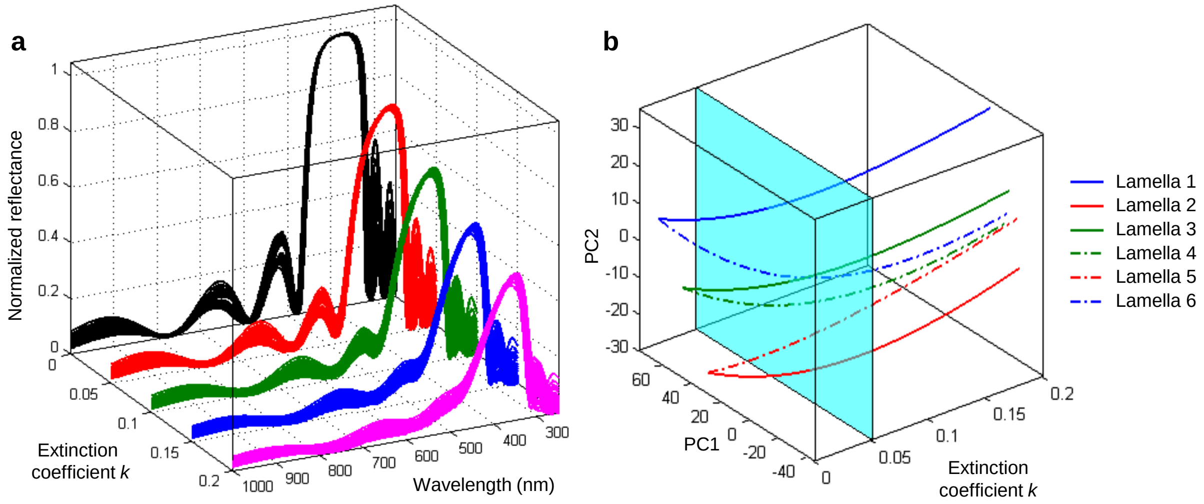

Supplementary Figure 2. Details of simulation results of vapour-response selectivity of bio-inspired six-lamella nanostructure with a variable extinction coefficient *k*. **(a)** Reflectance spectra of a six-lamellae nanostructure with k = 0, 0.05, 0.10, 0.15, and 0.20. Each spectral set at a given k has 55 spectra that correspond to three model vapours of n = 1.3, 1.4, 1.5, their three concentrations related to condensed liquid layers of thickness d = 5, 10, 15 nm, all for six lamella, and one blank (n = 1, d = 0 nm). **(b)** PCA scores plot summarizing effects of k = 0 – 0.2 on the resulting sensor selectivity via the effects of the contributions related to the refractive index of the vapour. Contributions of PCs: 56.9 and 22.7 %, for PC_1 and PC_2 respectively. Each line is an example of a single model vapour condition (n = 1.4, d = 10 nm), the trajectories of these lines provide visualization of the inter-lamellae response-selectivity as a function of increasing extinction coefficient. A vertical plane at k = 0.05 illustrates sensor resolution between all six lamella.

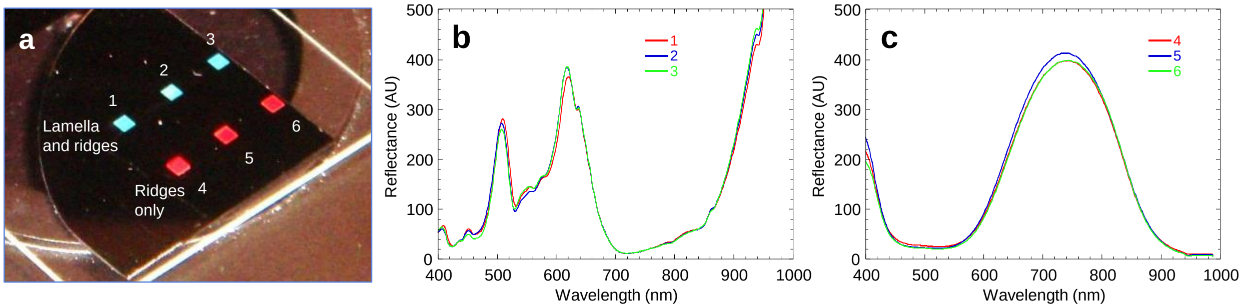

Supplementary Figure 3. Effect of lamella in fabricated nanostructures on reflectance spectra. **(a)** Reflected light image of six regions of photonic nanostructures fabricated with (regions 1 – 3) and without (regions 4 – 6) lamella. Each type of nanostructures was fabricated as three replicates (regions 1 – 3 and 4 – 6). **(b)** Reflectance spectra of photonic nanostructures with lamella (regions 1 – 3). Fabricated lamella produce complex reflectance pattern over the 450 – 700 nm spectral range. **(c)** Reflectance spectra of photonic nanostructures without lamella (regions 4 – 6). Structures without lamella produce a simple diffracted reflectance response with its maximum at ~ 740 nm. The area of structures in **(a)** is 2 mm x 2 mm.

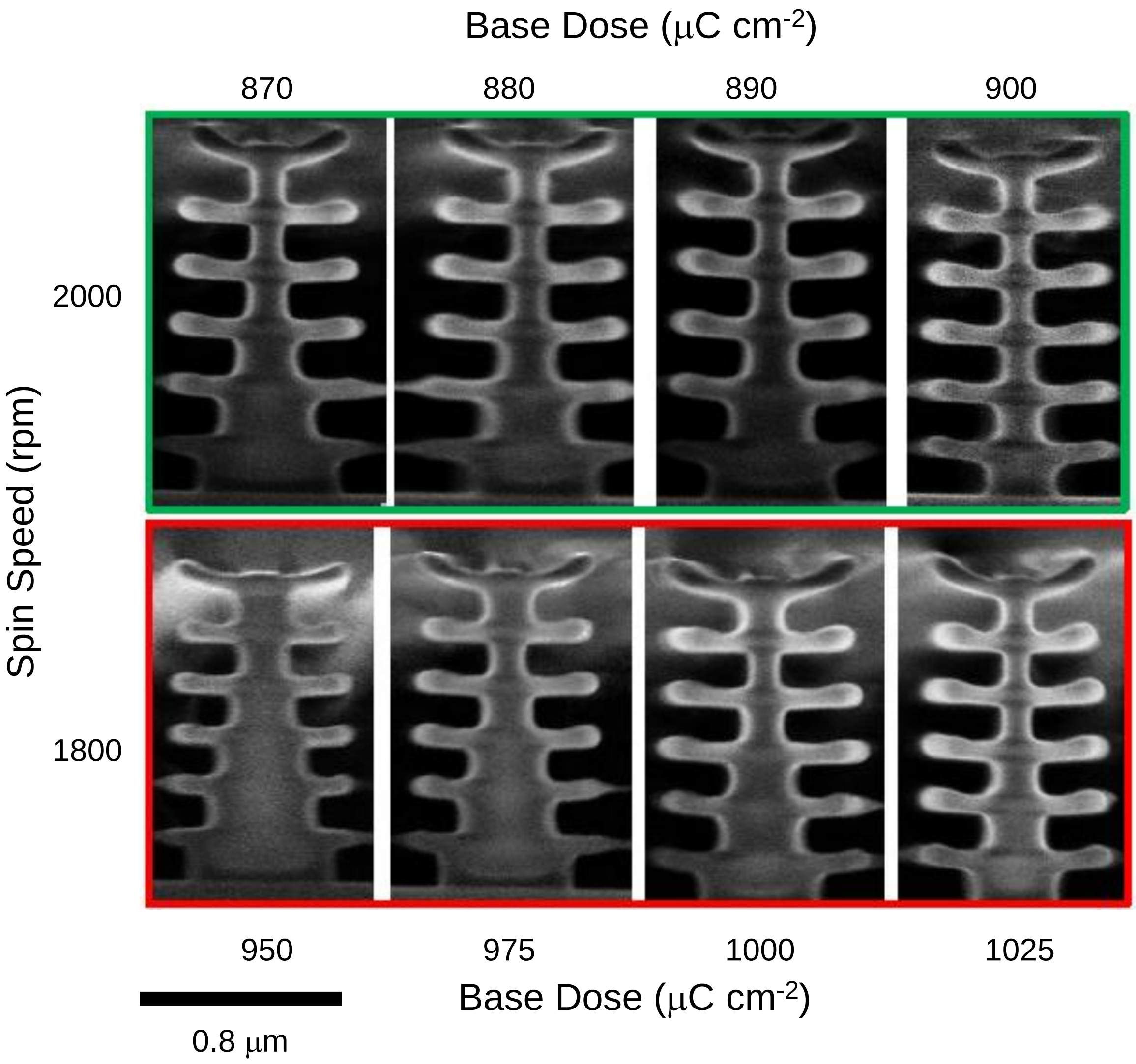

Supplementary Figure 4. Optimization of electron beam dose for the fabrication of nanostructures with six lamella. The dose was in the range from 950 to 1025 μC cm^{-2} for the structures formed using a 1800 rpm spin speed and was in the range from 870 to 900 μC cm^{-2} for the structures formed using a 2000 rpm spin speed. The optimal combination of the spin speed (2000 rpm) and electron beam dose (900 μC cm^{-2}) resulted in structures with the desired thinnest ridge.

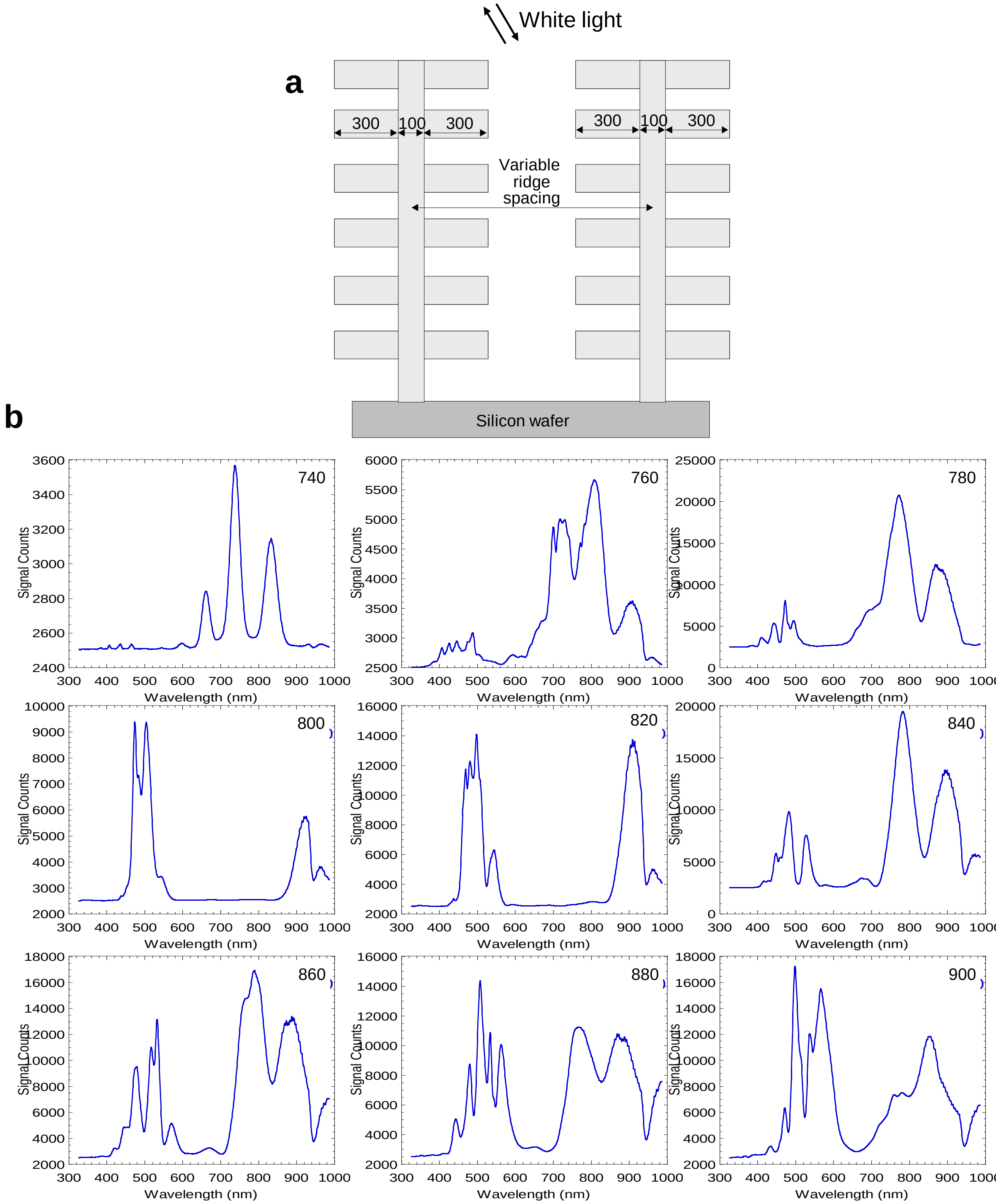

Supplementary Figure 5. Experimental observation of narrow resonances in six-lamella nanostructures fabricated with different ridge spacing. (a) Schematic and dimensions of fabricated nanostructures. Ridge spacing was varied from 740 to 900 nm in 20-nm steps keeping constant the length of the lamella (300 nm) and thickness of the ridge (100 nm). Measurements were done at 30 degrees to normal using a back-reflection fiber optic probe (see *Methods*). At 0 degrees reflectance was very simple without narrow resonances. **(b)** Reflectance spectra of nanostructures with variable ridge spacing. Numbers in the top right corner in each graph show ridge spacing in nm. Lamella were very poorly developed for 740 and 760 nm ridge spacing because of the insufficient gap between neighbor lamella.

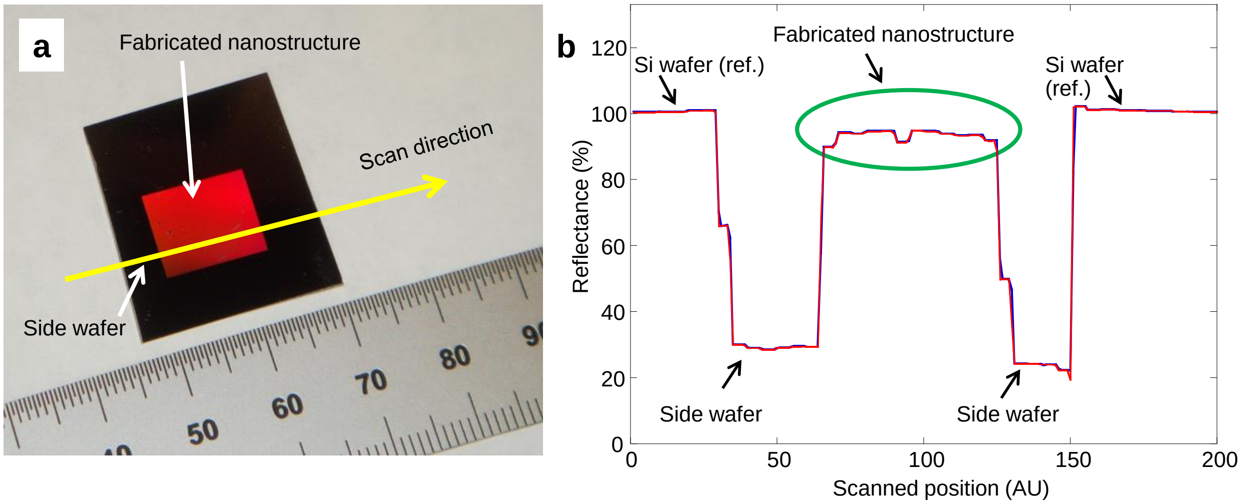

Supplementary Figure 6. Example of a fabricated large area nanostructure with a total area of >1 cm^2. (a) Reflected light photo of the fabricated large area nanostructure. **(b)** Results of determination of reflectance, measured normal to the surface with a bare Si water as a reference. Determined reflectance was 93.5 ± 1.5 % (mean ± 1 σ); RSD = 1.6 % as measured at 654 nm. A small reflectance dip in the middle of the scan is due to a slight indentation of the nanostructure upon handling.

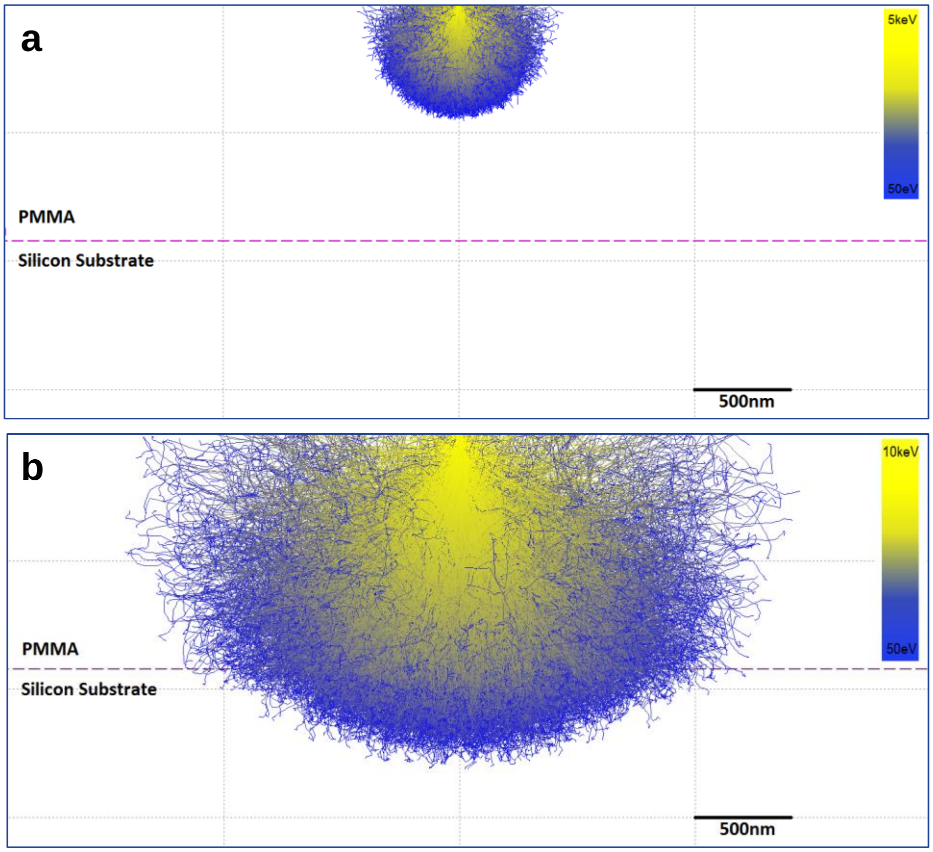

Supplementary Figure 7. Control of electron beam energy to produce a chemical surface gradient on the fabricated nanostructures. Results of simulations of electron beam penetration into the 1200-nm thick PMMA stack of lamella at (**a**) 5 keV and (**b**) 10 keV beam energies.

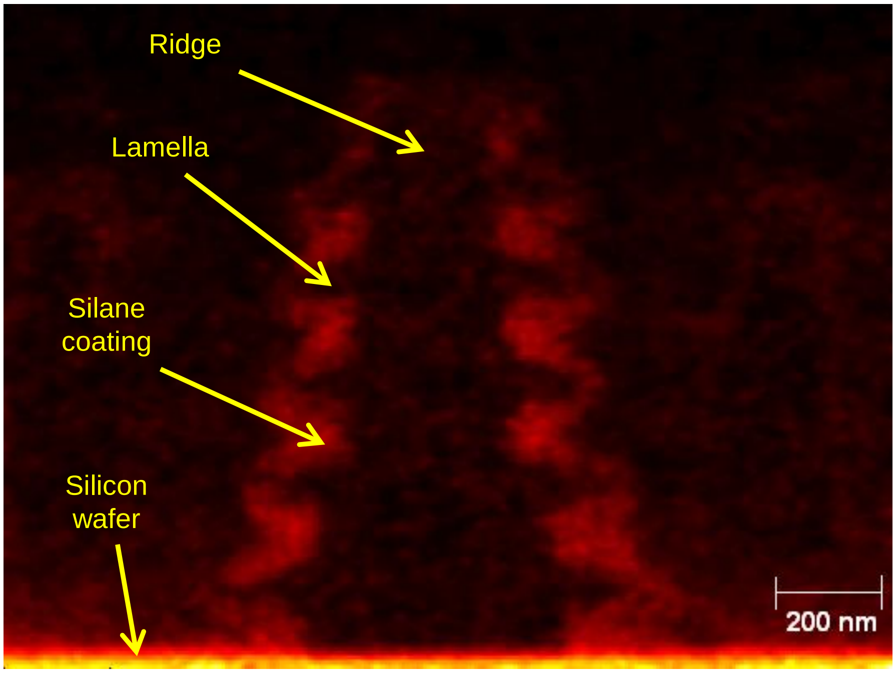

Supplementary Figure 8. Auger mapping of Si of a silane coating on the fabricated photonic nanostructure. Auger electron spectroscopy was performed on a cross-section prepared by using focused ion beam milling. The sample preparation by focused ion beam milling has slightly altered the geometry of the sample.

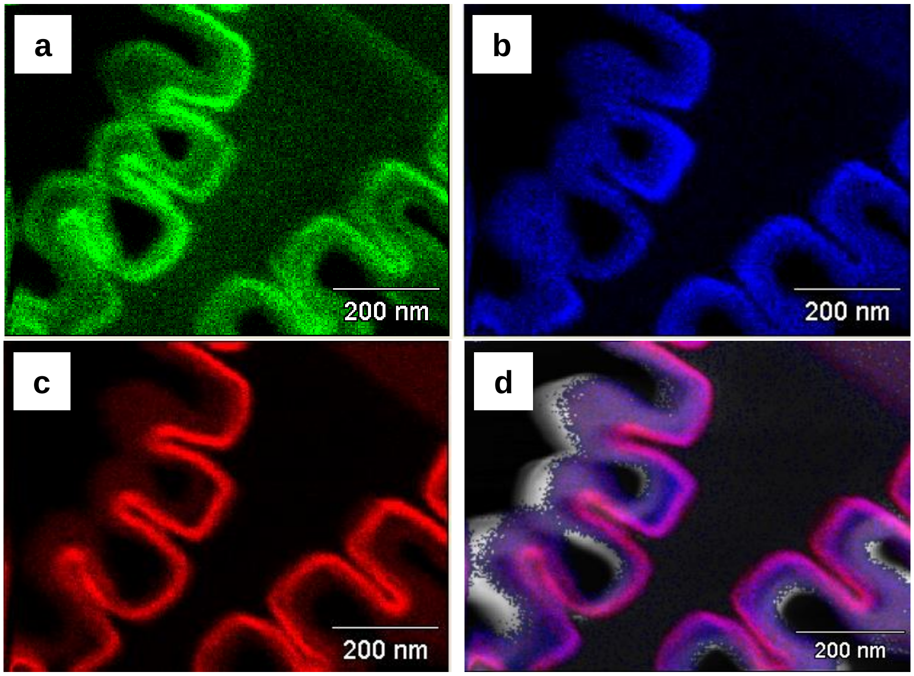

Supplementary Figure 9. Visualization of functional nonafluorohexyltrimethoxysilane coating on the photonic nanostructure upon focused ion beam milling for sample preparation. **(a – c)** X ray maps of O, F, and Al, respectively. **(d)** A combined X ray map. Sample preparation for the focused ion beam milling has slightly altered the geometry of the sample.

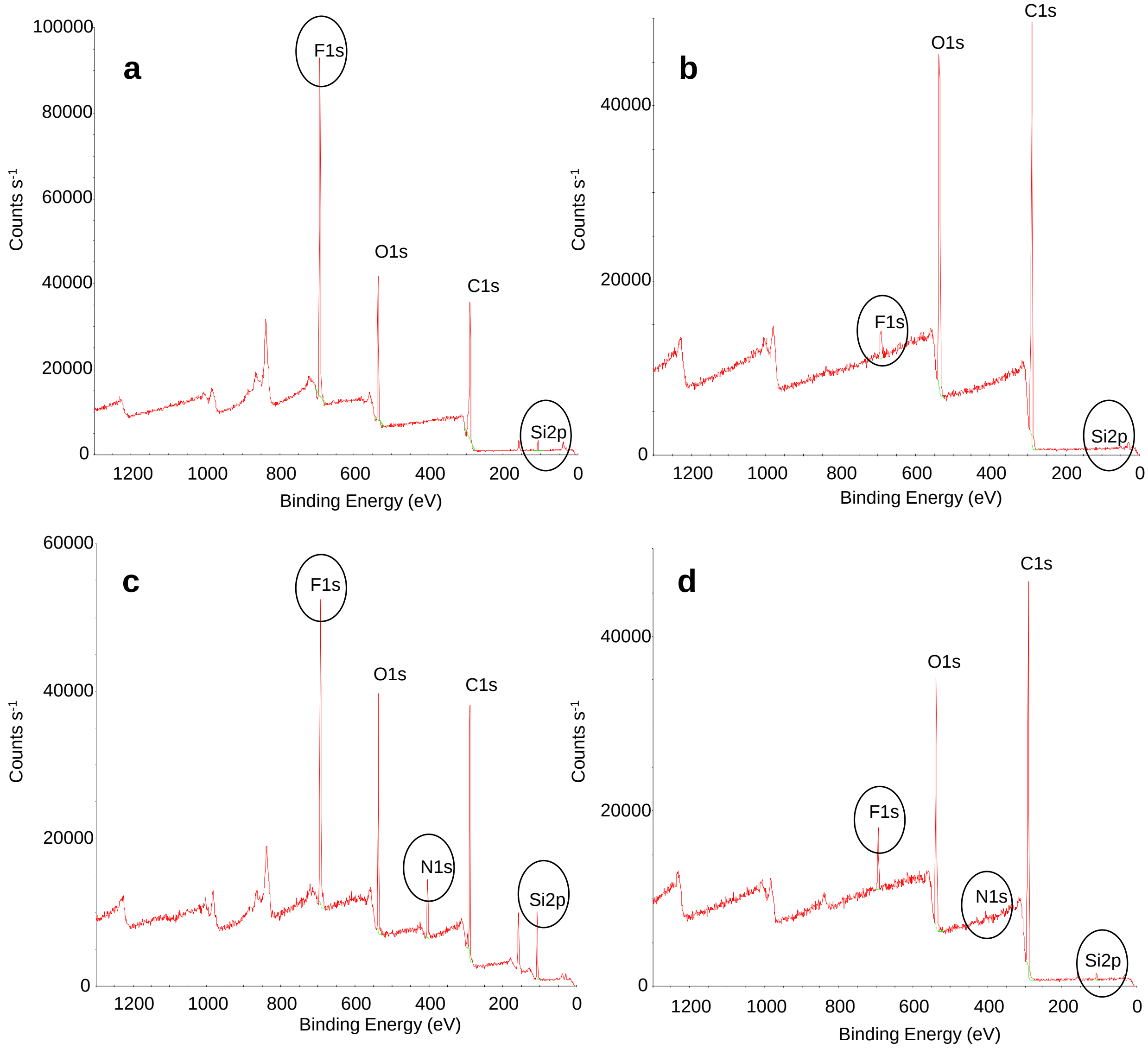

Supplementary Figure 10. Results of XPS analysis of fluorine-terminated and amine-terminated silanes on the photonic nanostructures. **(a, b)** Fluorine-terminated silane is nonafluorohexyltrimethoxysilane. **(c, d)** Amine-terminated silane is 3-aminopropyltrimethoxysilane. XPS spectra **(a, c)** before and **(b, d)** after electron beam exposure. Circled are atomic elements of most indicative to the presence of surface coating indicating a significant removal of the coating after the electron beam exposure.

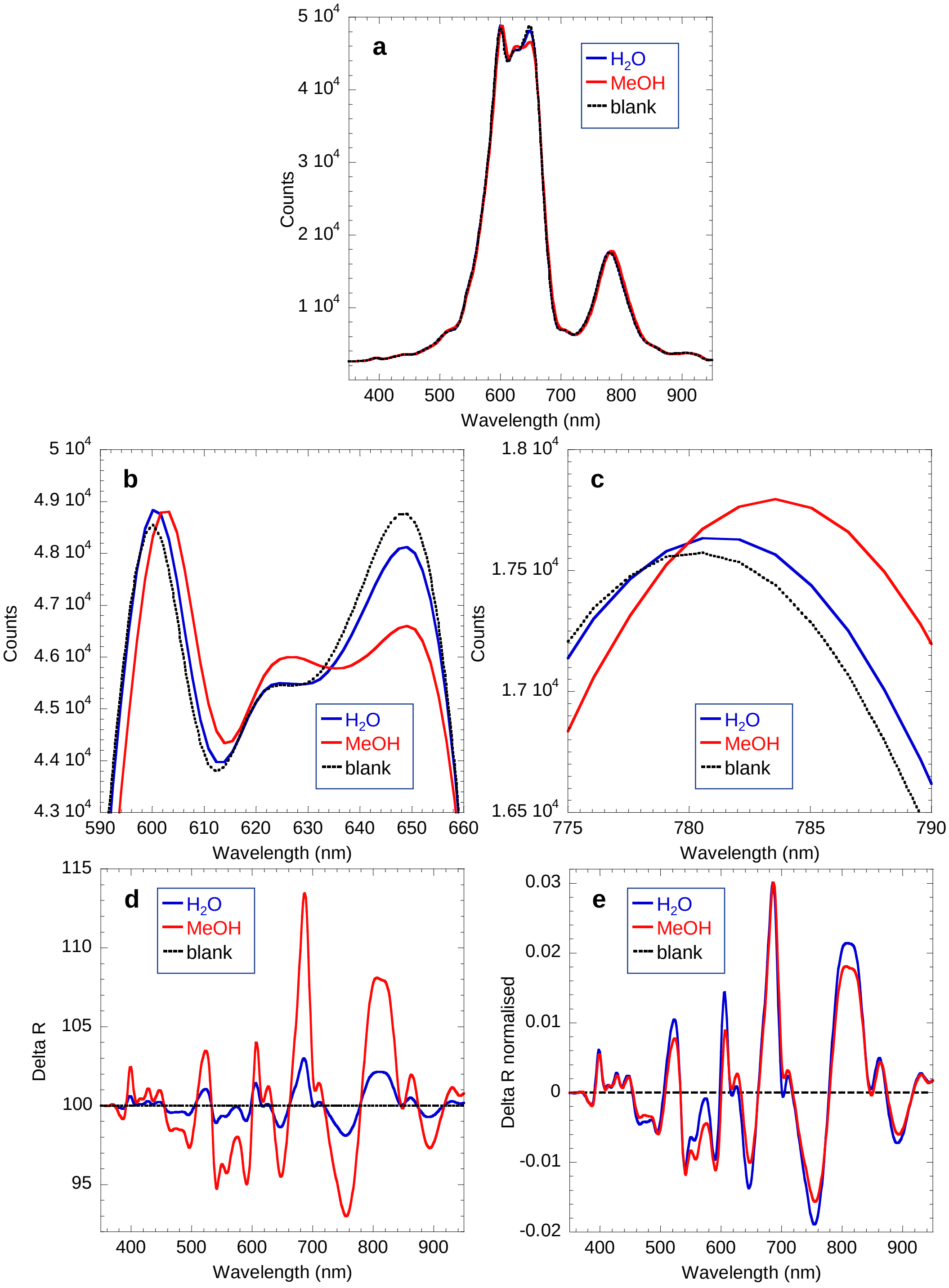

Supplementary Figure 11. Example of spectral reflectance of a fabricated nanostructure upon exposure to a dry carrier gas (blank) and exemplary individual vapours (methanol, MeOH and water, H_2O). (**a, b, c**) Spectra over different spectral ranges of the responses to the blank and to MeOH and H_2O vapours. In **a - c**, the Y axis is a raw spectrometer signal to indicate the magnitudes of the responses. (**d**) Differential reflectance spectra ΔR showing spectral details of responses of two individual vapours. (**e**) Differential reflectance spectra ΔR normalized by the peak at 687 nm showing relative spectral differences between two vapours. The concentration of vapours was 0.18 P/P_0, where P is vapour partial pressure and P_0 is the saturated vapour pressure.

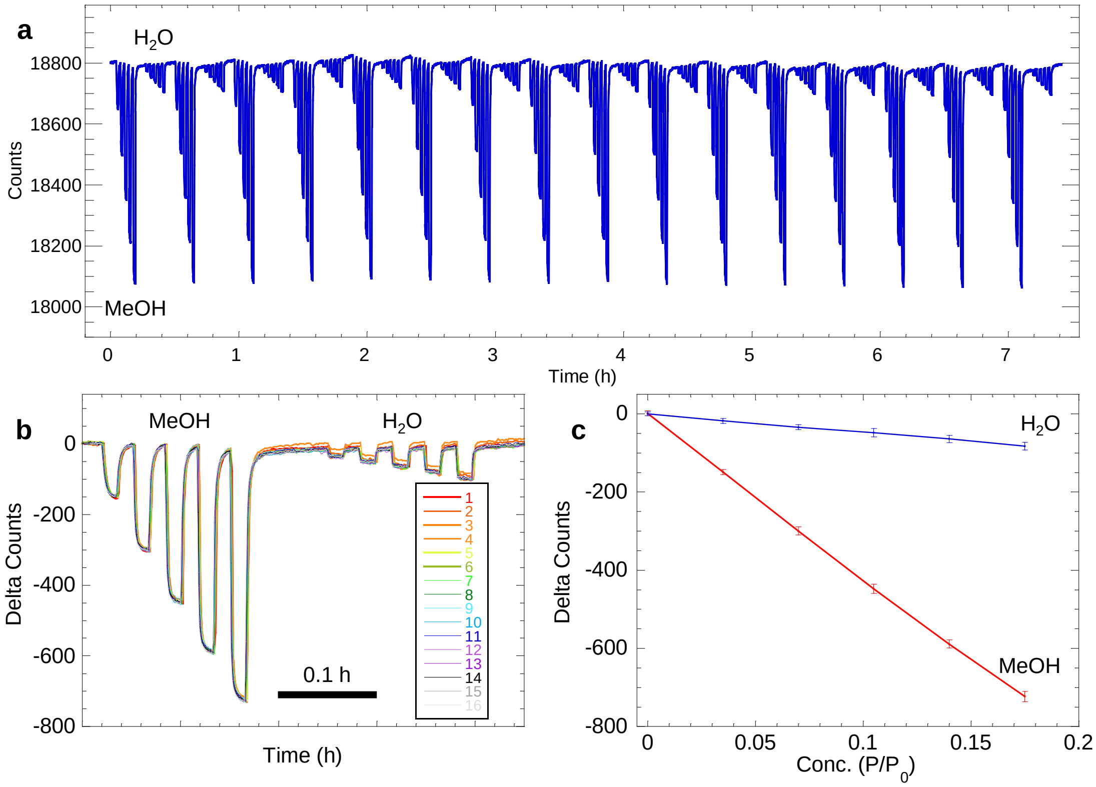

Supplementary Figure 12. Response stability and reproducibility of a fabricated sensing nanostructure upon exposures to 160 cycles of exemplary methanol (MeOH) and water (H_2O) vapours. (a) Response stability of raw sensor output: 0.04 % baseline drift at 562 nm over tested time of 7.5 h. (b) Response reproducibility to 80 cycles of MeOH and 80 cycles of H_2O. (c) Calibration curves for MeOH and H_2O. Measurement precision: 2 – 12 % relative standard deviation at the lowest tested concentrations of MeOH and H_2O. Each data point in (c) is the mean of measurements from 16 replicate cycles. Error bars = 3σ. Concentrations of vapours: 0.035, 0.07, 0.105, 0.14, and 0.175 P/P_0.

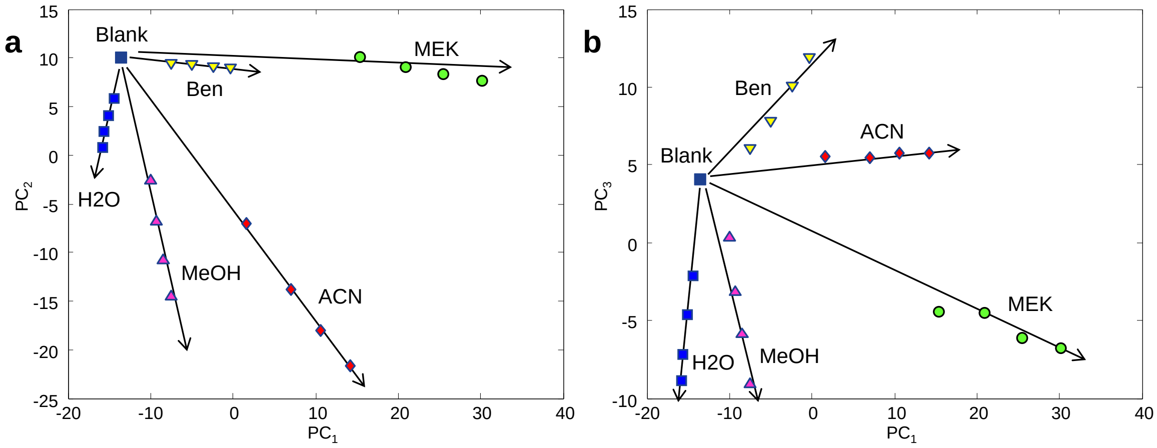

Supplementary Figure 13. Two-dimensional PCA scores plots demonstrating diversity of optical interactions of a nanofabricated FS-functionalized multivariable sensor with vapours of different nature. (a) PC_1 vs. PC_2 and **(b)** PC_1 vs. PC_3. Contributions of the first three PCs were 55.7, 30.3, and 12.1%, for PC_1 – PC_3 respectively. Vapours: benzene (Ben), acetonitrile (ACN), methyl ethyl ketone (MEK), methanol (MeOH), and water (H_2O). Concentrations of vapours: 0.05, 0.07, 0.09, and 0.11 P/P_0.

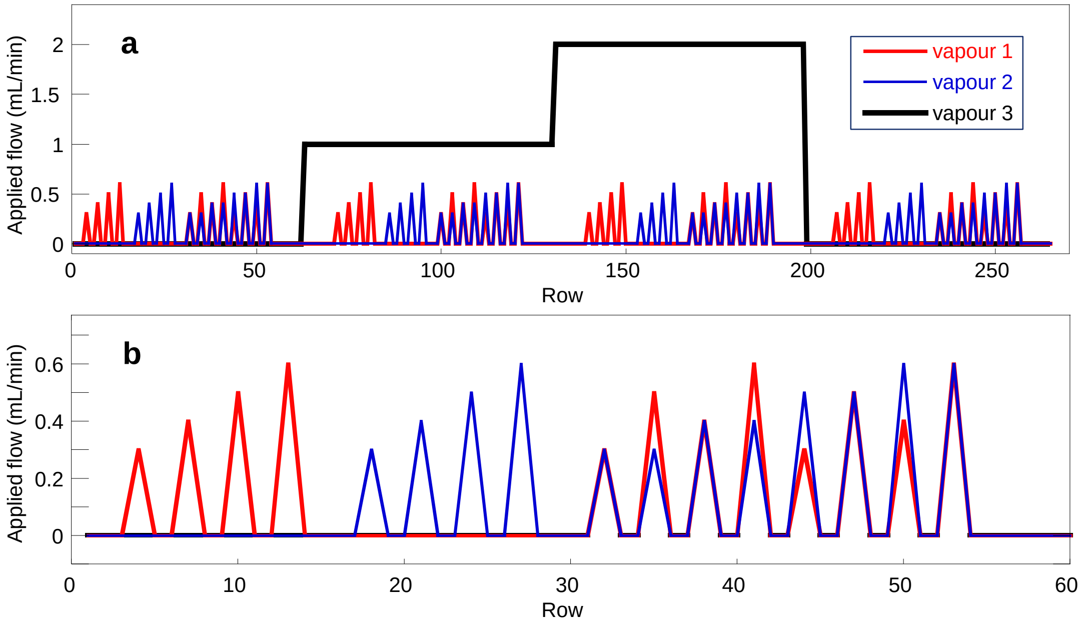

Supplementary Figure 14. Time-dependent sequence of concentrations of model analytes 1 and 2 (alcohols) and an interferent 3 (water) for testing of fabricated sensors, natural *Morpho* scales, and sensor arrays. (**a**) Concentration steps of vapours 1 – 3 and their mixtures. (**b**) Concentration steps of vapours 1 – 2 and their mixtures.

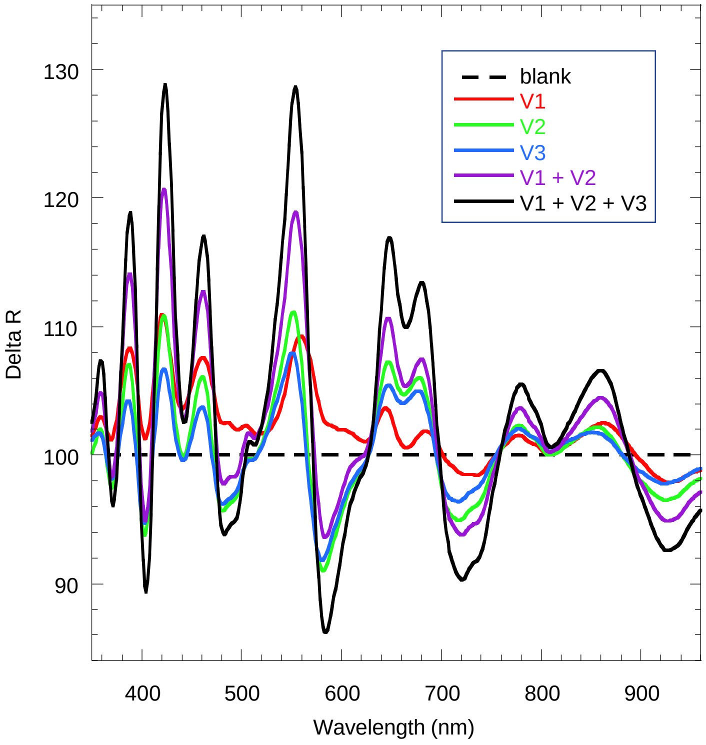

Supplementary Figure 15. *ΔR* spectra of a fabricated sensor upon exposure to methanol and propanol vapours, their binary mixture, and a ternary mixture with water vapour. Sensor: FS-functionalized, six-lamella structure. Vapours: V1, propanol, 0.11 P/P_0; V2, methanol, 0.11 P/P_0; and V3, water, 0.4 P/P_0. Blank, a dry carrier gas.

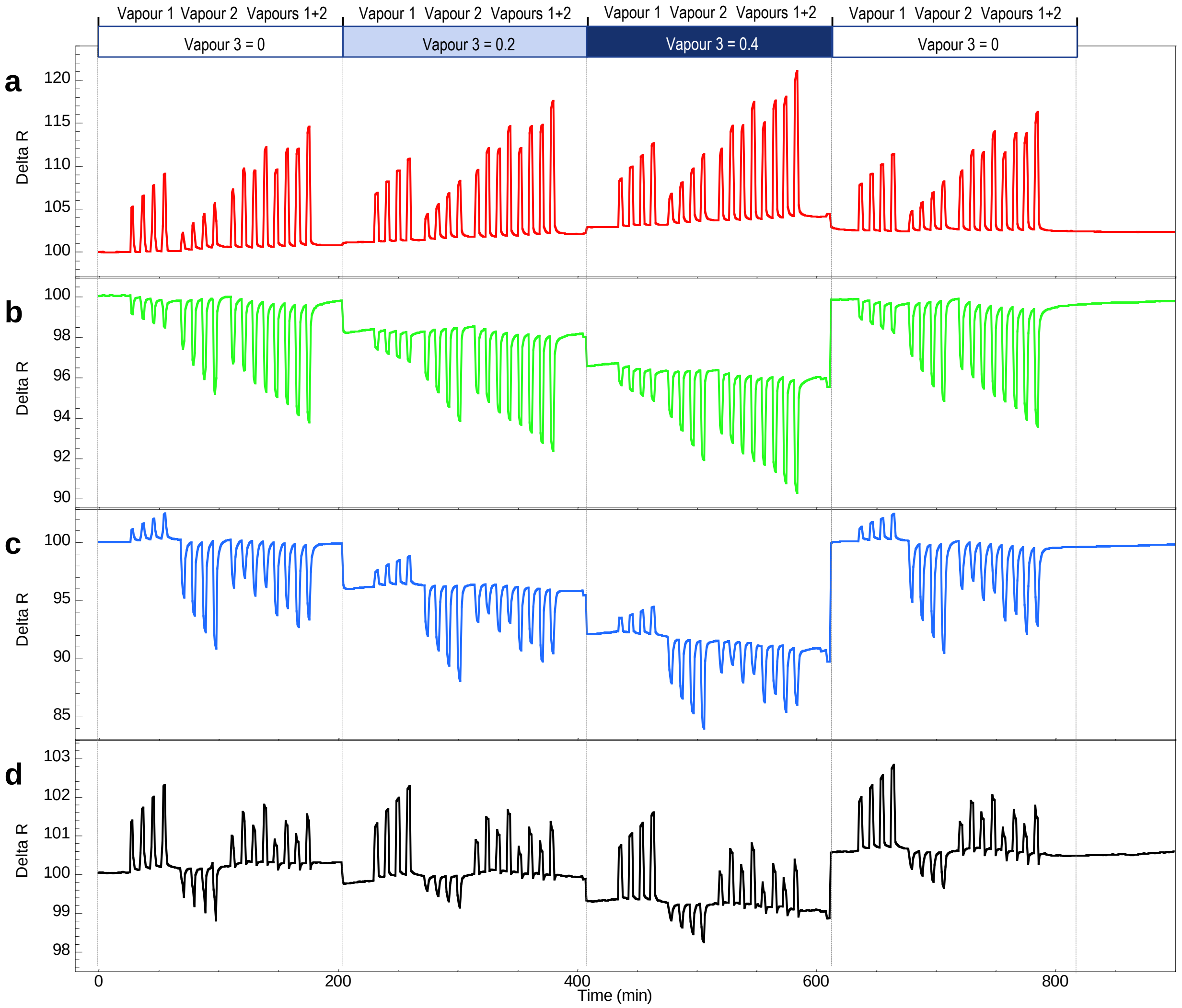

Supplementary Figure 16. A bio-inspired nanofabricated sensor detects individual methanol and propanol vapours and their mixtures in the presence of different levels of water background. Vapours 1 and 2 are model analyte vapours propanol and methanol, respectively. Vapour 3 is water as a model interferent. (**a – d**) *ΔR* sensor responses at 559, 723, 585, and 504 nm, respectively. (**a**) Minimal response to water vapour, vapours 1 - 3 produce responses in the same direction. (**b**) Strong additive response to water vapour, vapours 1 - 3 produce responses in the same direction. (**c**) Opposite response directions of vapours 1 and 2. Response magnitude of vapour 1 is only ~ 25% from the response magnitude of vapour 2. Responses to mixtures of vapours 1 and 2 are in the same direction as vapour 2. (**d**) Opposite response directions of vapours 1 and 2. Response magnitude of vapour 1 is ~ 200 % from the response magnitude of vapour 2. Responses to mixtures of vapours 1 and 2 are in the same direction as vapour 1. Dynamic responses upon propanol and methanol exposures have distinct recovery signatures at different wavelengths – e.g., fast and slow recovery at 559 and 723 nm, respectively and a non-monotonic recovery from propanol/methanol mixtures at 504 nm. These diverse dynamic signatures illustrate the existence of several interaction mechanisms of vapours with different regions of the functionalized nanostructure. Concentrations of vapours 1 and 2: 0.05, 0.07, 0.09, and 0.11 P/P_0. Concentrations of vapour 3: 0.2 and 0.4 P/P_0.

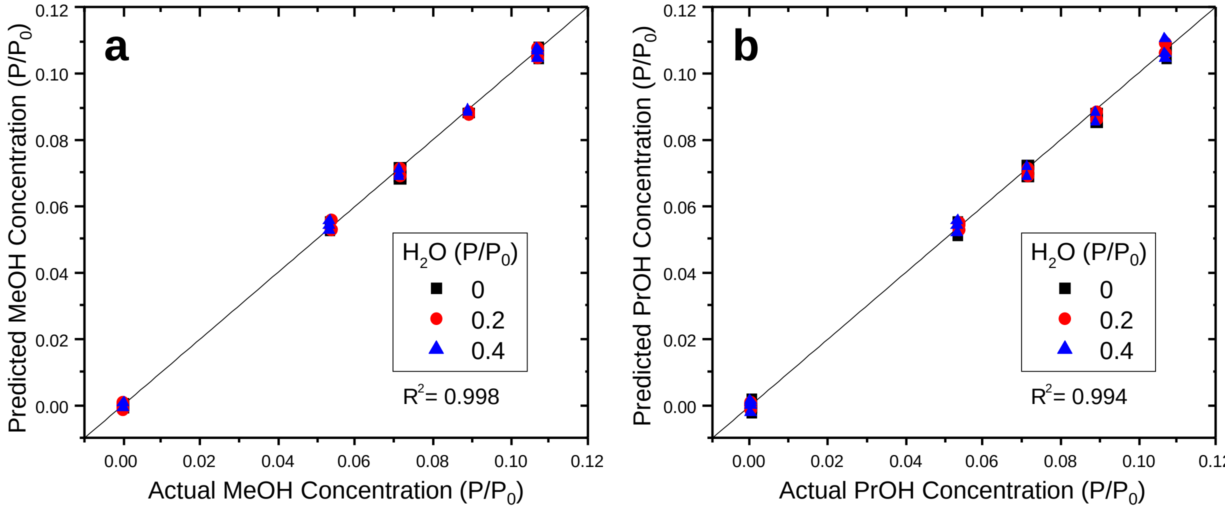

Supplementary Figure 17. An individual bio-inspired nanostructure quantifies individual methanol and propanol vapours and their mixtures in the presence of different levels of water vapour background. (**a, b**) Correlation plots between the actual and predicted concentrations of methanol and propanol vapours in the presence of different levels of background water vapour. Multivariate regression modeling using PLS technique was applied to quantify concentrations of individual vapours in these mixture experiments.

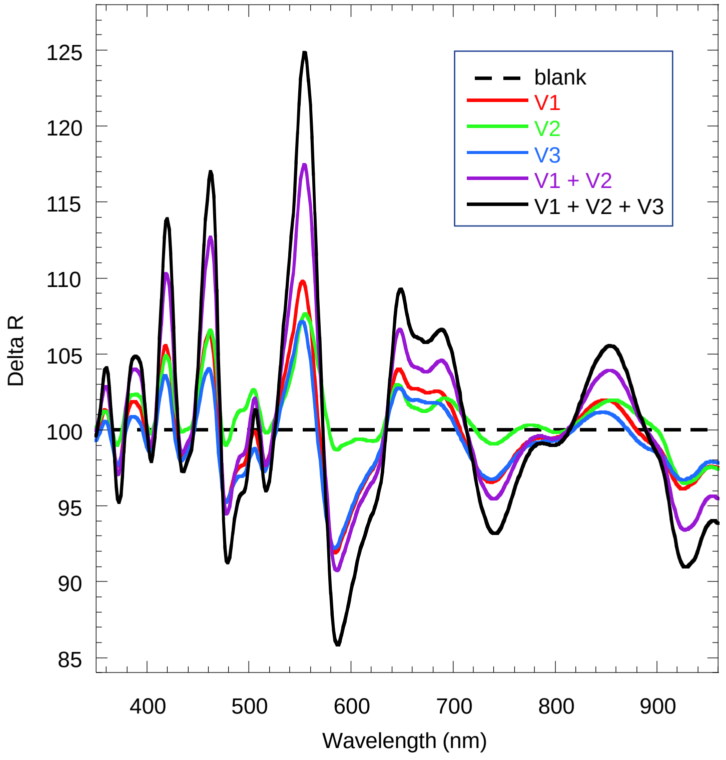

Supplementary Figure 18. *ΔR* spectra of a fabricated sensor upon exposure to methanol and ethanol vapours, their binary mixture, and a ternary mixture with water vapour. Sensor: FS-functionalized, six-lamella structure with microribs. Vapours: V1, methanol, 0.11 P/P_0; V2, ethanol, 0.11 P/P_0; V3, water, 0.4 P/P_0. Blank, a dry carrier gas.

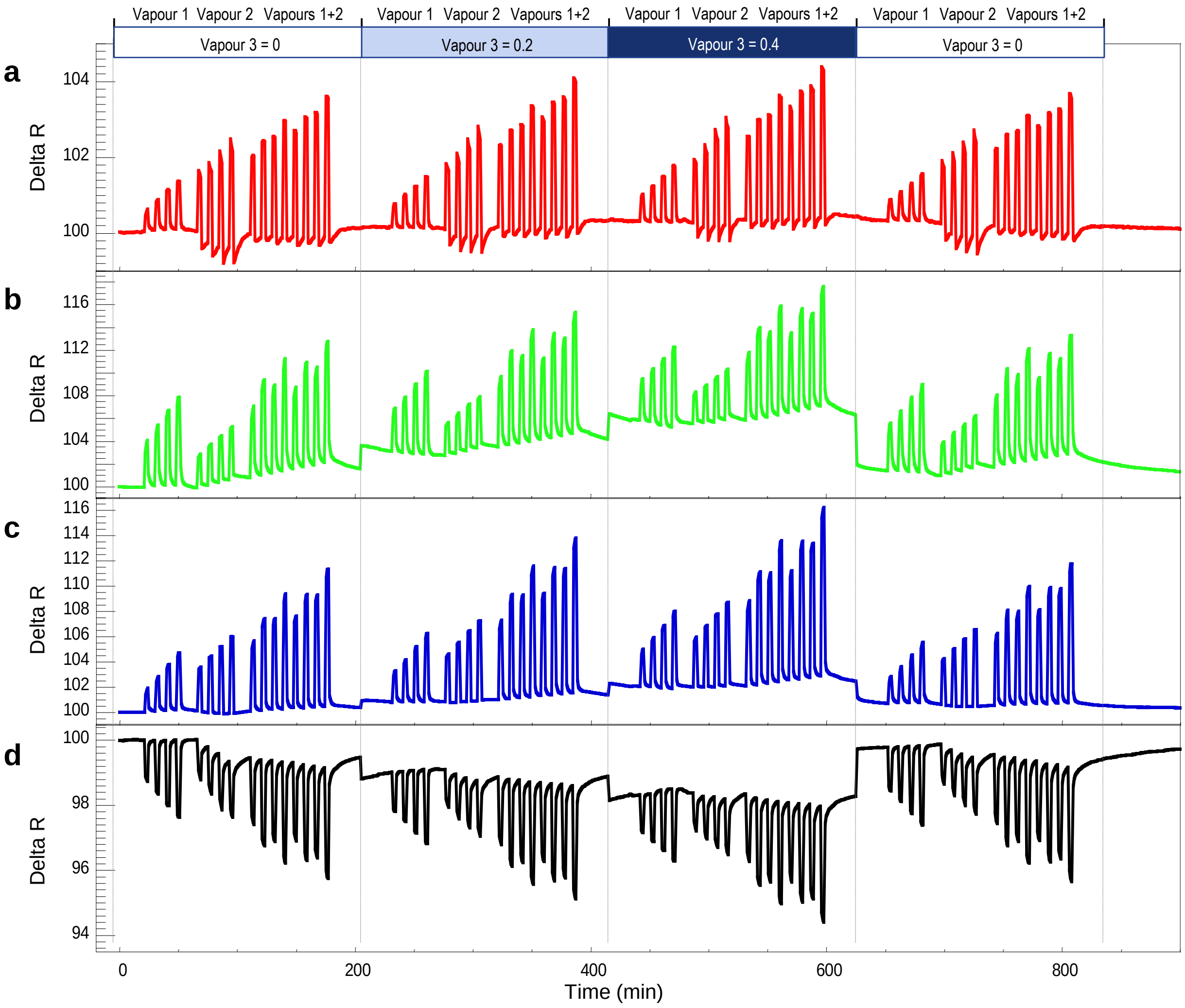

Supplementary Figure 19. A bio-inspired nanofabricated sensor detects individual methanol and ethanol vapours and their mixtures in the presence of different levels of water background. Vapours 1 and 2 are model analyte vapours methanol and ethanol, respectively. Vapour 3 is water as a model interferent. (**a – d**) ΔR sensor responses at 393, 546, 563, and 950 nm, respectively. (**a**) Minimal response to water vapour; vapour 1 has smaller response than vapour 2. (**b**) Strong additive response to water vapour; vapour 1 has larger response than vapour 2. (**c**) Weak additive response to water vapour; vapours 1 and 2 have similar responses. (**d**) Strong additive response to water vapour; vapour 1 has larger response than vapour 2. Concentrations of vapours 1 and 2: 0.05, 0.07, 0.09, and 0.11 P/P_0. Concentrations of vapour 3: 0.2 and 0.4 P/P_0.

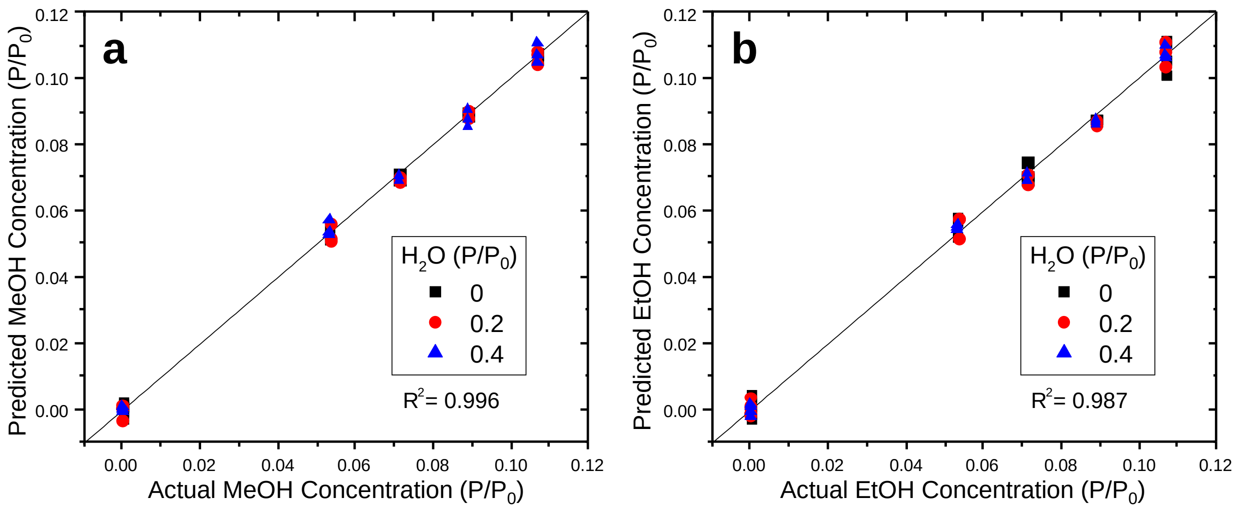

Supplementary Figure 20. An individual bio-inspired nanostructure quantifies individual methanol and ethanol vapours and their mixtures in the presence of different levels of water vapour background. (**a, b**) Correlation plots between the actual and predicted concentrations of methanol and ethanol vapours in the presence of different levels of background water vapour. Multivariate regression modeling using PLS technique was applied to quantify concentrations of individual vapours in these mixture experiments.

Supplementary Table 1. Ability of the FS-functionalized fabricated sensor for quantitation of individual vapours in mixtures demonstrated as the RMSEC and RMSECV values for methanol/propanol and methanol/ethanol vapour compositions in the presence of different levels of background water vapour (0, 0.2, and 0.4 P/P_0).

Mixtures of vapours	Quantified individual vapours	RMSEC (P/P_0)	RMSEC (ppm)	RMSECV (P/P_0)	RMSECV (ppm)
Methanol + propanol + water	Methanol	0.0010	167	0.0019	317
	Propanol	0.0016	44	0.0029	80
Methanol + ethanol + water	Methanol	0.0016	267	0.0028	468
	Ethanol	0.0034	265	0.0035	273

RMSEC = root mean square error of calibration
RMSECV = root mean square error of cross-validation

Supplementary Table 2. Contributions of PC_1 and PC_2 of PCA vapour-response models of natural *Morpho* structures, bio-inspired nanofabricated sensors, and conventional sensor arrays upon their exposures to analyte vapours and background.

Type of sensing system	Model analyte vapours	Background	PC_1 (%)	PC_2 (%)
Natural *Morpho* structure, FS-functionalized	methanol and propanol	water	79.12	17.66
Nanofabricated structure, FS-functionalized	methanol and propanol	water	68.92	30.39
Nanofabricated structure, bare	methanol and ethanol	water	99.39	0.35
Nanofabricated structure, FS-functionalized	methanol and ethanol	water	92.92	6.86
Sensor array, QCM	methanol and ethanol	water	99.98	0.02
Sensor array, MOX	methanol and ethanol	water	74.04	18.74

Supplementary Table 3. The extrapolated LODs for the tested exemplary vapours.

Exemplary vapours	Vapour pressure at 25 ^{o}C (Torr)	P (ppm) at 0.05 P/P_0	% ΔR at 0.05 P/P_0	Extrapolated LOD (ppm)
Benzene	94.8	6237	2.1	45
Acetonitrile	88.8	5842	9.5	9
Methyl ethyl ketone	90.6	5961	12.6	7
Methanol	127	8355	9.6	13
Water	23.8	1566	2.9	8
Ethanol	59.3	3901	6.1	10
Propanol	21	1382	7.6	3

LOD = limit of detection

Supplementary Note 1. Effects of fabrication tolerances

To evaluate performance variability of developed nanostructured multivariable sensors, their fabrication tolerances should be determined. Tolerances related to the physical structure will control the reproducibility of the reflectance spectra, while tolerances related to structure functionalization will control vapour-selectivity. Common methodologies of determination of fabrication tolerances of photonic nanostructures will be implemented.[1-3] Calibration will be performed as a batch- or individual-sensor calibration, dependent on the required sensor accuracy. Calibration will include development of transfer functions of sensor responses for vapours at identified environmental conditions. The determined fabrication tolerances will aid the development of the robust transfer functions.

Supplementary Note 2. Differential reflectance spectral response $\Delta R(\lambda)$

Existing photonic nanostructured sensors exhibit a significant color change only at relatively high concentrations of detected vapours.[4,5] When the spectral shifts are relatively small, the differential reflectance spectral response $\Delta R(\lambda)$ of a sensor is measured before and after analyte exposure, as[5]

$$\Delta R(\lambda) = 100\% \text{ x } \{R(\lambda)/R_0(\lambda)\}, \qquad \text{(Eq. 1)}$$

where $R(\lambda)$ and $R_0(\lambda)$ are sensor spectra upon exposure to an analyte and a blank carrier gas, respectively. Thus, the common features in the two spectra before and after vapour exposure cancel and the $\Delta R(\lambda)$ spectrum accentuates the subtle differences due to vapour response.[6]

Supplementary Note 3. Sensor ability for quantitation of individual vapours in mixtures

Recently, the limit of recognition (LOR) has been introduced[33-35] as a criterion for evaluating sensor arrays and defined as the concentration below which a vapour can no longer be recognized from its response pattern. To take into the account not only vapour recognition but also vapour quantitation in mixtures, two more conservative estimations may be appropriate. Such estimations are the root mean square error of calibration (RMSEC) and the root mean square error of cross-validation (RMSECV) obtained from a multivariate regression model that provides the ability to recognize and quantify analytes in the presence of interferences.[36] For example, multivariate PLS models are a standard tool in quantitation of analytes in mixtures in numerous industrial applications that utilize Process Analytical Technology methodologies.[37] The errors of concentration values of analytes obtained using a multivariate PLS model account for all statistical noise and are very conservative in order to provide robust quantitation.

We have built two PLS models to describe responses of the developed sensors to (1) methanol/propanol and (2) methanol/ethanol vapour compositions in the presence of three levels of background water vapour. Supplementary Figs. 17 and 20 depict correlation plots between the actual and predicted concentrations of model analyte vapours in these experiments. The RMSEC and RMSECV values for methanol/propanol and methanol/ethanol vapour compositions when blended with a variable moisture background are presented in Supplementary Table 1. In this work, we have not focused to minimize RMSEC or RMSECV values but focused instead on selectivity enhancement of individual sensors. "Discussion" section of the main text outlines our approach toward increasing of sensor signal.

Supplementary Note 4. Vapour selectivity of natural *Morpho* structures, bio-inspired nanofabricated sensors, and conventional sensor arrays

The first two PCs were indicative of the main dispersion directions[7,8] upon exposure to two individual vapours (see Supplementary Table 2). For methanol and propanol exposures, the contribution of PC_2 for *Morpho* and nanofabricated structures were 18% and 30%, respectively. For methanol and ethanol exposures, the contribution of PC_2 for bare nanofabricated structure and QCM sensor array were < 1%, while for the MOX sensor array it was 19% and for the FS-functionalized nanofabricated structure it was 7%. Although the MOX sensor array had the response dispersion larger than that of the FS-functionalized nanofabricated structure, the latter had a better ability to operate at variable humidity without significant non-linearity effects (see Fig. 6 and Supplementary Figs. 16, 19). The prediction ability of the developed sensors to quantify vapours in their mixtures is illustrated in Supplementary Figs. 17 and 20, Supplementary Table 1, and Supplementary Note 3.

Supplementary Note 5. Simplification of fabricated vs. natural *Morpho* nanostructures

The choice for symmetric lamella was driven to simplify sensor fabrication, in contrast to a tapered butterfly wing structure formed during butterfly growth.[9] The needs for a symmetric lamella width and optical loss in lamella material were two independent requirements in the bio-inspired sensor. The slight loss in the fabricated nanostructure was found to be needed to induce controlled differences in the reflectance spectra from the top and bottom regions of the multilayer lamella stacks. A broadband loss is already present in chitin material of the natural butterfly structure but it only reduces reflectance strength; it is melanin pigment layer underneath the photonic structures that results in a blue-color reflectance of certain *Morpho* butterflies.[9]

Supplementary Note 6. Limit of detection of the sensor

The limit of detection (LOD) is an important sensor parameter that is defined as analyte concentration yielding a signal equal three standard deviations of the blank measurement:[6]

$$\text{LOD} = 3\sigma_{\Delta R}\ [\text{vapour}] / S, \qquad \text{(Eq. 2)}$$

where $\sigma_{\Delta R}$ is the standard deviation of the blank measurement (measurement noise), measured in the ΔR mode as % of reflectance change as compared to the blank carrier gas (Eq. 1), [vapour] is the smallest measured vapour concentration, and S is the sensor signal magnitude at [vapour].

Sensor responses S to vapours were determined at their ΔR spectral peaks at ~400 – 900 nm that provided the largest magnitudes. The smallest $\sigma_{\Delta R}$ was obtained with the spectrograph integration time of 5 ms, averaging 200 samples per saved spectrum, taking spectra for 5 min, and making these measurements in triplicate. The resulting standard deviation of the blank was $\sigma_{\Delta R} = 0.005 \pm 0.001$. The mean value $\sigma_{\Delta R} = 0.005$ was utilized for calculation of LOD.

Since the focus of this study was on sensor selectivity, the smallest concentrations of vapours were at 0.05 P/P_0. Therefore, we consider calculated LODs only as initial extrapolations, being ~100 – 1,000 times smaller than [vapour]. These extrapolated LODs for exemplary vapours are presented in Supplementary Table 3. The differences of 200 – 10,000 times between calculated LODs and [vapour] are common in initial tests of new promising sensors.[10-12] In our upcoming studies focused on optimization of these sensors for specific applications, a more detailed vapour testing will be performed with vapour concentrations down to 0.001 – 0.01 P/P_0.

As shown in Eq. 2, the LOD can be improved (minimized) by two equally important steps: by increasing the sensor signal and/or by reducing the measurement noise. "Discussion" section of the main text outlines our approach toward increasing of sensor signal. At the same

time, measurement noise can be reduced by a number of common methods. For example, one of the methods utilizes dual-beam measurements that improved the LOD of a porous silicon sensor from ppm to ppb levels.[13,14] Another method involves a dynamic correction of a spectrograph dark spectrum that reduced the noise to $5x10^{-6}$ absorbance units,[15] corresponding to $\sigma_{\Delta R} = 0.001$.

Known photonic resonant vapour sensors operate on univariate vapour quantitation principles based either on the detection of wavelength shift of the resonance peak or signal intensity change. These sensors are based on porous silicon, self-assembled colloidal particles, mesoporous photonic crystals, inverse opals, and high-Q resonators as analyzed in recent reviews.[16-27] The LODs of porous silicon,[28] self-assembled colloidal particles,[29] mesoporous photonic crystals,[30] inverse opals,[31] and hybrid plasmonic-photonic crystals[32] sensors are in the single or double-digit ppm range when measurements are performed using a straightforward spectral data collection with a laboratory spectrometer and a white light source.

Supplementary References

1. Hasar, U. C., Ozbek, I. Y., Oral, E. A., Karacali, T. & Efeoglu, H. The effect of silicon loss and fabrication tolerance on spectral properties of porous silicon Fabry-Perot cavities in sensing applications. *Opt. Express* **20**, 22208-22223 (2012).
2. Kraus, S. & Lucki, M. Structural tolerances of optical characteristics in various types of photonic lattices. *Adv. Electr. Electron. Eng.* **12**, 245-251 (2014).
3. Chavelas, D., Oikonomou, P., Botsialas, A., Argitis, P., Papanikolaou, N., Goustouridis, D., Beltsios, K., Lidorikis, E., Raptis, I. & Chatzichristidi, M. Lithographically tuned one dimensional polymeric photonic crystal arrays. *Optics and Laser Technology* **68**, 105112 (2015).
4. Li, Y. Y., Cunin, F., Link, J. R., Gao, T., Betts, R. E., Reiver, S. H., Chin, V., Bhatia, S. N. & Sailor, M. J. Polymer Replicas of Photonic Porous Silicon for Sensing and Drug Delivery Applications. *Science* **299**, 2045-2047 (2003).
5. Potyrailo, R. A., Ghiradella, H., Vertiatchikh, A., Dovidenko, K., Cournoyer, J. R. & Olson, E. Morpho butterfly wing scales demonstrate highly selective vapour response. *Nature Photonics* **1**, 123-128 (2007).
6. Ingle, J. D., Jr. & Crouch, S. R. *Spectrochemical Analysis* (Prentice Hall, Englewood Cliffs, NJ, 1988).
7. Palacios, M. A., Nishiyabu, R., Marquez, M. & Anzenbacher Jr., P. Supramolecular chemistry approach to the design of a high-resolution sensor array for multianion detection in water. *J. Am Chem. Soc.* **129**, 7538-7544 (2007).
8. Lim, S. H., Feng, L., Kemling, J. W., Musto, C. J. & Suslick, K. S. An Optoelectronic Nose for the Detection of Toxic Gases. *Nature Chemistry*, 562-567 (2009).
9. Ghiradella, H. Light and color on the wing: structural colors in butterflies and moths. *Appl. Opt.* **24**, 3492-3500 (1991).
10. Li, J., Lu, Y., Ye, Q., Cinke, M., Han, J. & Meyyappan, M. Carbon nanotube sensors for gas and organic vapor detection. *Nano Lett.* **3**, 929-933 (2003).
11. Snow, E. S., Perkins, F. K. & Robinson, J. A. Chemical vapor detection using single-walled carbon nanotubes. *Chem. Soc. Rev.* **35**, 790-798 (2006).
12. Feng, L., Musto, C. J., Kemling, J. W., Lim, S. H. & Suslick, K. S. A colorimetric sensor array for identification of toxic gases below permissible exposure limits. *Chem. Comm.* **46**, 2037-2039 (2010).
13. Gao, J., Gao, T. & Sailor, M. J. Porous-silicon vapor sensor based on laser interferometry. *Appl. Phys. Lett.* **77**, 901-903 (2000).
14. Gao, J., Gao, T., Yang Li, Y. & Sailor, M. J. Vapor sensors based on optical interferometry from oxidized microporous silicon films. *Langmuir* **18**, 2229-2233 (2002).

15. Dahlin, A. B., Tegenfeldt, J. O. & Höök, F. Improving the instrumental resolution of sensors based on localized surface plasmon resonance. *Anal. Chem.* **78**, 4416-4423 (2006).
16. Sailor, M. J. Color me sensitive: Amplification and discrimination in photonic silicon nanostructures. *ACS Nano* **1**, 248-252 (2007).
17. Zhao, Y., Xie, Z., Gu, H., Zhu, C. & Gu, Z. Bio-inspired variable structural color materials. *Chem. Soc. Rev.* **41**, 3297-3317 (2012).
18. Xu, H., Wu, P., Zhu, C., Elbaz, A. & Gu, Z. Z. Photonic crystal for gas sensing. *J. Mater. Chem. C* **1** 6087-6098 (2013).
19. Burgess, I. B., Lončar, M. & Aizenberg, J. Structural colour in colourimetric sensors and indicators. *J. Mater. Chem. C* **1**, 6075-6086 (2013).
20. Pacholski, C. Photonic crystal sensors based on porous silicon. *Sensors* **13**, 4694-4713 (2013).
21. Harraz, F. A. Porous silicon chemical sensors and biosensors: A review. *Sens. Actuators B* **202**, 897-912 (2014).
22. Wang, F., Meng, Z., Xue, F., Xue, M., Lu, W., Chen, W., Wang, Q. & Wang, Y. Responsive photonic crystal for the sensing of environmental pollutants. *Trends Environm. Anal. Chem.* **3-4** (2014).
23. Fenzl, C., Hirsch, T. & Wolfbeis, O. S. Photonic crystals for chemical sensing and biosensing. *Angew. Chem. Int. Ed.* **53**, 3318-3335 (2014).
24. Yetisen, A. K., Naydenova, I., Da Cruz Vasconcellos, F., Blyth, J. & Lowe, C. R. Holographic sensors: Three-dimensional analyte-sensitive nanostructures and their applications. *Chem. Rev.* **114**, 10654-10696 (2014).
25. Baker, J. E., Sriram, R. & Miller, B. L. Two-dimensional photonic crystals for sensitive microscale chemical and biochemical sensing. *Lab on a Chip* **15**, 971-990 (2015).
26. Fan, X. (ed.) *Advanced Photonic Structures for Biological and Chemical Detection* (Springer, New York, NY, 2009).
27. Luchansky, M. S. & Bailey, R. C. High-Q optical sensors for chemical and biological analysis. *Anal. Chem.* **84**, 793-821 (2012).
28. Ruminski, A. M., King, B. H., Salonen, J., Snyder, J. L. & Sailor, M. J. Porous silicon-based optical microsensors for volatile organic analytes: Effect of surface chemistry on stability and specificity. *Adv. Funct. Mater.* **20**, 2874-2883 (2010).
29. Yang, H., Jiang, P. & Jiang, B. Vapor detection enabled by self-assembled colloidal photonic crystals. *J. Colloid Interface Sci.* **370**, 11-18 (2012).
30. Xie, Z., Cao, K., Zhao, Y., Bai, L., Gu, H., Xu, H. & Gu, Z. Z. An optical nose chip based on mesoporous colloidal photonic crystal beads. *Adv. Mater.* **26**, 2413-2418 (2014).
31. Zhang, Y., Qiu, J., Hu, R., Li, P., Gao, L., Heng, L., Tang, B. Z. & Jiang, L. A visual and organic vapor sensitive photonic crystal sensor consisting of polymer-infiltrated SiO2 inverse opal. *Phys. Chem. Chem. Phys.* **17**, 9651-9658 (2015).
32. Yu, X., Shi, L., Han, D., Zi, J. & Braun, P. V. High quality factor metallodielectric hybrid plasmonic-photonic crystals. *Adv. Funct. Mater.* **20**, 1910-1916 (2010).
33. Zellers, E. T., Park, J., Hsu, T. & Groves, W. A. Establishing a limit of recognition for a vapor sensor array. *Anal. Chem.* **70**, 4191-4201 (1998).
34. Hsieh, M.-D. & Zellers, E. T. Limits of Recognition for Simple Vapor Mixtures Determined with a Microsensor Array. *Anal. Chem.* **76**, 1885-1895 (2004).
35. Jin, C. & Zellers, E. T. Limits of recognition for binary and ternary vapor mixtures determined with multitransducer arrays. *Anal. Chem.* **80**, 7283-7293 (2008).
36. Otto, M. *Chemometrics: Statistics and Computer Application in Analytical Chemistry* (Wiley-VCH, Weinheim, Germany, 1999).
37. Bakeev, K. A. *Process Analytical Technology: Spectroscopic Tools and Implementation Strategies for the Chemical and Pharmaceutical Industries* (Wiley, Chichester, United Kingdom, 2010).
